# Global patterns in genomic diversity underpinning the evolution of insecticide resistance in the aphid crop pest *Myzuspersicae*

**DOI:** 10.1038/s42003-021-02373-x

**Published:** 2021-07-07

**Authors:** Kumar Saurabh Singh, Erick M. G. Cordeiro, Bartlomiej J. Troczka, Adam Pym, Joanna Mackisack, Thomas C. Mathers, Ana Duarte, Fabrice Legeai, Stéphanie Robin, Pablo Bielza, Hannah J. Burrack, Kamel Charaabi, Ian Denholm, Christian C. Figueroa, Richard H. ffrench-Constant, Georg Jander, John T. Margaritopoulos, Emanuele Mazzoni, Ralf Nauen, Claudio C. Ramírez, Guangwei Ren, Ilona Stepanyan, Paul A. Umina, Nina V. Voronova, John Vontas, Martin S. Williamson, Alex C. C. Wilson, Gao Xi-Wu, Young-Nam Youn, Christoph T. Zimmer, Jean-Christophe Simon, Alex Hayward, Chris Bass

**Affiliations:** 1 College of Life and Environmental Sciences, Biosciences, University of Exeter, Penryn, Cornwall UK; 2 Departamento de Entomologia e Acarologia, Escola Superior de Agricultura “Luiz de Queiroz,”, Universidade de São Paulo, Piracicaba, Brazil; 3 Department of Crop Genetics, John Innes Centre, Norwich Research Park, Norwich, UK; 4 INRAE, UMR 1349 IGEPP, Le Rheu, France; 5 Departamento de Producción Vegetal, Universidad Politécnica de Cartagena, Cartagena, Spain; 6 Department of Entomology and Plant Pathology, North Carolina State University, Raleigh, NC USA; 7 Laboratory of Biotechnology and Nuclear Technologies, National Center of Nuclear Sciences and Technologies, Biotechpole of Sidi Thabet, Sidi Thabet, Ariana Tunisia; 8 Department of Biological and Environmental Sciences, University of Hertfordshire, Hatfield, UK; 9 Instituto de Ciencias Biológicas, Universidad de Talca, Talca, Chile; 10 Boyce Thompson Institute, Ithaca, NY USA; 11 Department of Plant Protection at Volos, Institute of Industrial and Fodder Crops, Hellenic Agricultural Organization ‘DEMETER’, Volos, Greece; 12 Department of Sustainable Crop Production, Section Sustainable Crop and Food Protection, Università Cattolica del Sacro Cuore, Piacenza, Italy; 13 Bayer AG, Crop Science Division, R&D, Monheim, Germany; 14 Tobacco Research Institute, Chinese Academy of Agricultural Sciences, Qingdao, China; 15 Scientific Center of Zoology and Hydroecology, National Academy of Science, Republic of Armenia, Yerevan, Armenia; 16 Cesar, Parkville, Victoria Australia; 17 School of BioSciences, The University of Melbourne, Parkville, Victoria Australia; 18 The Department of General Ecology and Methods of Biology Teaching, Belarusian State University, Minsk, Republic of Belarus; 19 Institute of Molecular Biology & Biotechnology, Foundation for Research & Technology Hellas, Crete, Greece; 20 Department of Crop Science, Agricultural University of Athens, Athens, Greece; 21 Department of Biointeractions and Crop Protection, Rothamsted Research, Harpenden, UK; 22 Department of Biology, University of Miami, Coral Gables, FL USA; 23 Department of Entomology, College of Plant Protection, China Agricultural University, Beijing, China; 24 Department of Applied Biology, College of Agricultural and Life Science, Chungnam National University, Daejeon, Korea; 25 Present Address: Syngenta Crop Protection, Werk Stein, Schaffhauserstrasse, Stein, Switzerland

**Keywords:** Evolutionary genetics, Agricultural genetics

## Abstract

The aphid *Myzus persicae* is a destructive agricultural pest that displays an exceptional ability to develop resistance to both natural and synthetic insecticides. To investigate the evolution of resistance in this species we generated a chromosome-scale genome assembly and living panel of >110 fully sequenced globally sampled clonal lines. Our analyses reveal a remarkable diversity of resistance mutations segregating in global populations of *M. persicae*. We show that the emergence and spread of these mechanisms is influenced by host–plant associations, uncovering the widespread co‐option of a host-plant adaptation that also offers resistance against synthetic insecticides. We identify both the repeated evolution of independent resistance mutations at the same locus, and multiple instances of the evolution of novel resistance mechanisms against key insecticides. Our findings provide fundamental insights into the genomic responses of global insect populations to strong selective forces, and hold practical relevance for the control of pests and parasites.

## Abstract (web summary)

Singh, Bass and colleagues generate new genomic resources for the aphid crop pest, *Myzus persicae*, comprising a chromosome-scale genome assembly and resequenced genomes of >100 globally sampled clonal lines. Using this dataset, they uncover both mechanisms underpinning the evolution of insecticide resistance and ecological factors that influence its emergence and spread.

## Introduction

Insect pests damage agricultural production, endanger food security, and transmit diseases that harm crop plants, livestock, and humans. Although pesticides provide an important tool for controlling crop pests and disease vectors, a wide range of pest species have repeatedly shown the capacity to overcome them through the evolution of resistance^1–3^. In many cases, resistance now represents the single greatest threat to the sustainability of insect pest control^1,2,4^. Consequently, new strategies and tools to combat resistance, underpinned by a greater understanding of the ecological and evolutionary processes involved, are urgently required. In this battle against pesticide resistance recent technological advances in genome sequencing provide promise that a new era of research, employing powerful genomic interrogation of global insect populations, can provide new insight into the molecular and evolutionary response of pests to selection^1,5^.

Aphids are hemipteran insects that are of particular applied importance as plant pests, causing tens of millions to billions US$ of yield loss per annum across a wide range of food and commodity crops^6^. Aphids are also exceptional models for the study of a range of fundamental ecological and evolutionary topics, including reproductive mode variation, insect-plant interactions, virus transmission, phenotypic plasticity, symbiosis, and insecticide resistance^2,7^. Research on this important group of insects has been greatly facilitated by the publication of draft genome sequence assemblies for a number of aphid species^7–11^. However, to date, studies of genetic variation within aphid populations have primarily used a limited number of molecular markers or candidate gene-based approaches^12,13^.

One of the most economically important aphid crop pests worldwide, and an emerging insect research model, is the peach potato aphid, *Myzus persicae* (Sulzer). The status of this species as a pest is enhanced by its global distribution, remarkable efficiency as a vector of more than 100 different plant viruses, and its extremely broad host range^14^. Indeed, the exceptional ability of *M. persicae* to colonise over 100 plant species from 40 diverse families suggests that it is a rare example of a true generalist^8^. This contrasts with other aphid species which tend to specialise on a limited number of hosts^6^, and/or consist of several host-adapted biotypes / races, that specialise on a subset of the total host range^13^. While clonal lineages of *M. persicae* can colonise distantly related host species in the laboratory^8,15^, certain races that have adapted to feed on tobacco can be morphologically and genetically differentiated from *M. persicae sensu stricto* (s.s.), and have been formally named as *M. persicae subsp. nicotianae*^16,17^. This clearly suggests that host races/subspecies do form in *M. persicae sensu latu* (s.l.), however, whether this is true for other host plants, and the impact of this on gene flow and genetic differentiation is poorly understood.

In common with many invertebrates, *M. persicae* acts as a host for mutualistic symbionts. Like other aphids, *M. persicae* feeds on the phloem sap of plants and thus relies on the intracellular mutualistic bacterium *Buchnera aphidicola* to provide essential amino acids that are missing or rare in its diet. In addition to its obligate association with *B. aphidicola*, *M. persicae* may develop facultative associations with additional bacterial symbionts that can provide other ecological benefits^18^. However, to date, the frequency of these secondary symbionts in populations of *M. persicae* worldwide remains unknown.

The control of *M. persicae* worldwide has relied almost exclusively on the use of synthetic insecticides, and this has led to the evolution of resistance to multiple classes of chemistry^2^. At least seven independent mechanisms of resistance have been described in this species to date, including mutation of insecticide targets in the aphid nervous system, enhanced expression of insecticide detoxifying enzymes and reduced penetration of insecticide through the cuticle (reviewed in^2^). However, the ecological and evolutionary factors influencing the emergence and spread of these mechanisms in global populations of *M. persicae* has never been investigated. *M. persicae* has also evolved mechanisms to overcome natural insecticides, such as the secondary metabolites produced by plants. The best example of this is the tobacco-adapted subspecies, *M. p*. *nicotianae*, that exhibits high levels of resistance to nicotine, the potent natural insecticide produced by this plant^19^.

The extent of resistance in *M. persicae* currently represents a major threat to its sustainable control, with just a handful of insecticides retaining efficacy^2^. Thus, it is increasingly important to understand the underlying processes and mechanisms involved in the evolution of resistance to older and recently deployed compounds, to guide the development of effective strategies to prolong the life of current and future insecticides. Here, we addressed this need by generating a population genomic resource for *M. persicae* comprising a high-quality chromosomal-scale genome assembly together with resequenced genomes of 127 clonal aphid lines collected from all continents where crops are grown. We leverage this combination of fine-scale genome-wide data with large-scale sampling across geographic and host divides, to investigate both the mechanisms underpinning insecticide resistance, and ecological and evolutionary factors influencing its emergence and spread. Specifically, our analyses of this population genomic dataset addressed the following key questions:

1. Based on the aphid clones sequenced, to what extent are *M. persicae* populations structured by geography and/or host plant association?
2. How does observed population structure influence the emergence and spread of insecticide resistance genes, and is there evidence for the co-option of host plant adaptations during the evolution of resistance to synthetic insecticides?
3. How repeatable is resistance evolution: (i) To what extent is resistance a consequence of single versus multiple alternative resistance mutations? (ii) Do resistance mutations typically arise once and spread, or have multiple independent origins?
4. Can we leverage our new genomic and biological resources to uncover novel resistance to recently introduced insecticides and characterise the underpinning genetic architecture?

## Results and discussion

### Generation of genomic resources for *M. persicae*

**Fig. 1:**
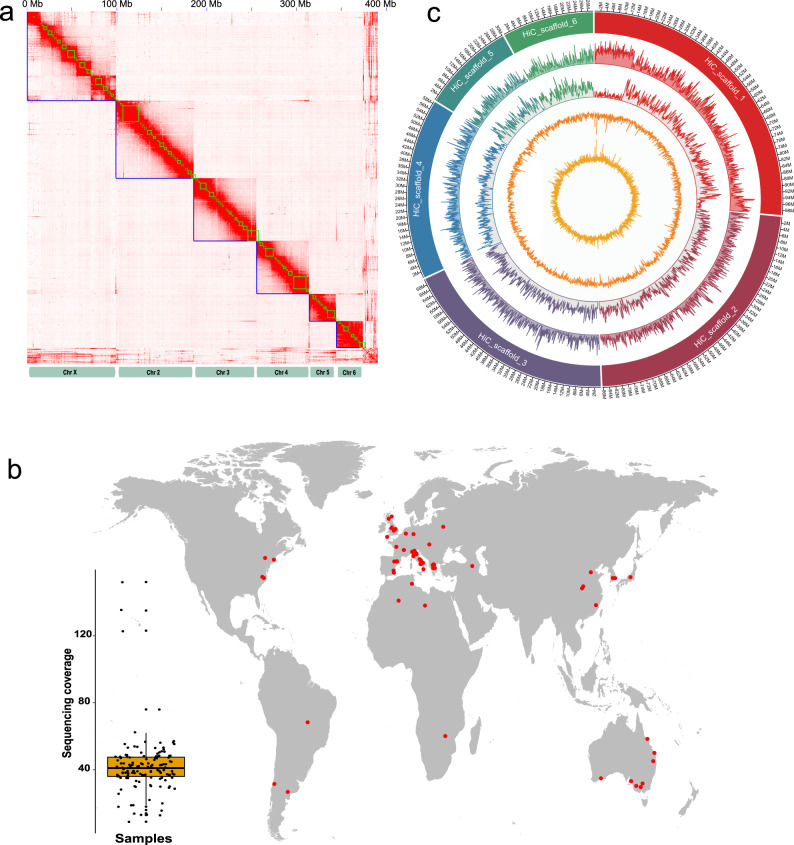
New biological and genomic resources for the aphid *Myzus persicae* reveal the genome-wide patterns of genetic variation in clones sampled from across the world. **a** Chromosome-scale genome assembly of *M. persicae* clone G006. Heatmap shows frequency of HiC contacts along the genome assembly. Blue lines indicate super scaffolds and green lines show contigs, with the X axis showing cumulative length in millions of base pairs (Mb). **b** Geographic origin and sequence coverage of the 127 resequenced clones of *M. persicae* used in this study. **c** Circular plot of genome-wide genetic variation in a global sample of 127 *M. persicae* clones. The outermost circle represents the 6 chromosome-sized super-scaffolds of the genome assembly, with scaffold 1 the X chromosome. Moving inwards the circles represent: gene density, SNP density, GC and AT% over 100 kb non-overlapping windows.

To enhance the accuracy of population genomic and genome-wide association studies in *M. persicae*, we generated a chromosome-level genome assembly of the *M. persicae* s.s. clone G006^20^. Almost 40 Gb of PacBio single-molecule real-time (SMRT) sequencing data were assembled into 773 contigs, with an N50 of 3,162,279 bp. These contigs were then categorized and ordered into six chromosome-scale scaffolds, corresponding to the haploid chromosome number of this species^21^, using in vivo chromatin conformation capture (HiC) data (Fig. 1a). This resulted in a final assembly of 391 Mb, with 95.7% of assembled content contained in the six scaffolds, and a scaffold N50 of 69.9 Mb (Supplementary Table 1). DNAseq data derived from *M. persicae* males and asexual females were used to identify the X chromosome (scaffold 1), as described previously^9^. The completeness of the gene space in the assembled genome was assessed using the Benchmarking Universal Single-Copy Orthologues (BUSCO) pipeline^22^, with 97.3% of the Arthropoda test gene set found to be present as complete single copies (Supplementary Table 1). Thus, the new G006 assembly (G006v2) represents a near complete and highly contiguous assembly and a significant improvement on the existing short read assembly of this clone (G006v1)^8^ (Supplementary Table 1). Structural genome annotation using a workflow incorporating RNAseq data predicted a total of 23,214 protein-coding genes in the assembly. Of these, 21,899 were successfully assigned functional annotations based on BLAST searches against the non-redundant protein database of NCBI and the InterPro database.

To generate a population genomic resource for aphid research we assembled a collection of 127 clones of *M. persicae s.l*. derived from 19 countries covering all continents except Antarctica (Fig. 1b, Supplementary Data 1). The clones were collected from 14 host plants encompassing a range of agriculturally important crops. Of the assembled clones, ~110 are maintained as asexual lineages in the Bass laboratory. These are available to other researchers as live cultures or preserved material, providing an excellent resource for genotype–phenotype association studies. All clones were sequenced using Illumina paired-end sequencing to an average coverage of 40X (Fig. 1b, Supplementary Data 1), with data mapped to the reference chromosome-level assembly of G006v2, and a collection of >30 reference genomes of known insect symbionts and a number of viruses known to infect aphids^23,24^ (see methods).

We identified a total of 45,627,645 high-quality biallelic single nucleotide polymorphisms (SNPs) comprising 14,647,103 non-reference-homozygous and 30,980,542 heterozygous variant calls. This represents, on average, one variant every 9 bp of the single copy genome of *M. persicae*. For each clone, variant density was similar across the five autosomes averaging between 3.2 and 3.6 SNPs every 1 kb of genomic length (Fig. 1c). SNP density was lower on the X chromosome (2.8 SNPs every kb, Fig. 1c), which may reflect the greater purifying selection of deleterious recessive alleles on X in hemizygous (X0) males^25^. Variants were distributed evenly across the autosomes, however, a marked reduction in SNP frequency was observed towards the ends of the X chromosome (Hi-C Scaffold 1, Fig. 1c), which contain a high density of repetitive DNA^9^. Pairwise comparisons of genetic distance between the sequenced clones (Supplementary Data 2) identified two clones from the US, S75 and S126, as most similar, with 101 and 92 unique variant sites respectively, and clone S6 from the UK and clone S107 from the US the most divergent, with 35,365 and 59,698 unique variant sites respectively.

Metagenomic analysis of the microbial component present in the sequence data revealed that, as expected, all clones carry the primary symbiont *Buchnera aphidicola* (Supplementary Data 3). With respect to other microbes, while several of the clones were found to be infected with the densovirus MpDNV, they were found to be essentially free of secondary symbionts (Supplementary Data 3). This finding suggests that secondary symbionts may play a less important role in enhancing the (context-dependent) fitness of *M. persicae* when compared to other aphid species^26^.

### Geography and host plant influence *M. persicae* population structure

**Fig. 2:**
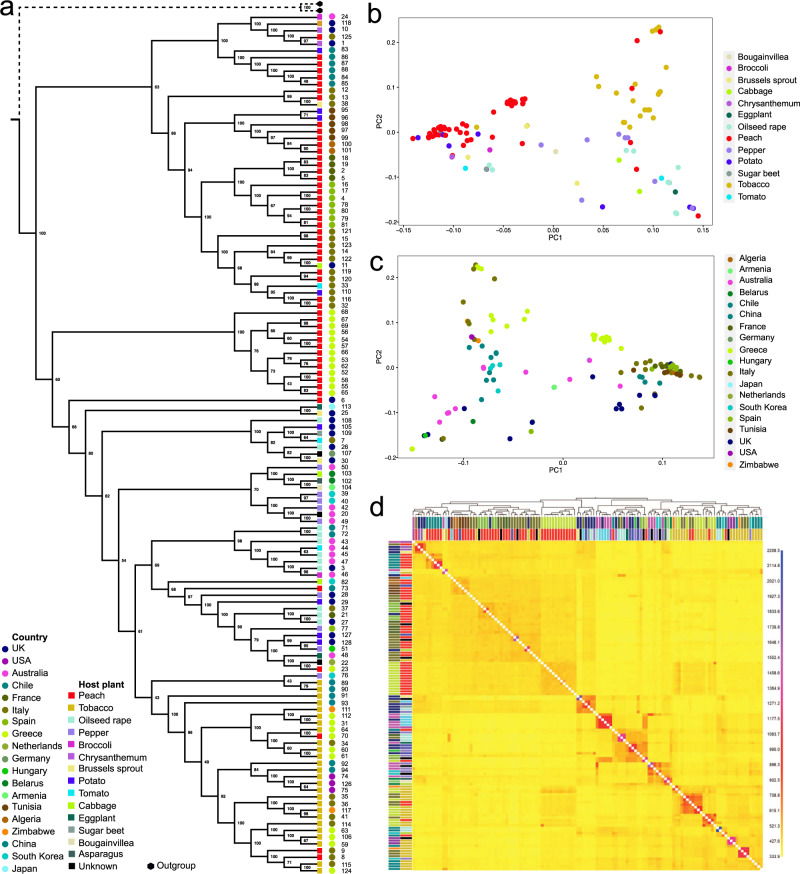
Phylogenetic relationship and population structure of 127 *M. persicae* clones. **a** Maximum likelihood phylogeny based on >1 M biallelic SNPs. Data from two samples of *Myzus cerasi* were used as an outgroup. The geographic origin of clones and the host plant from which they were collected are indicated by coloured circles and squares respectively. Clone identification numbers (corresponding to Supplementary Data 1) are also included. For a representation of the tree as a phylogram see Fig. S1. **b**, **c** Principal component analysis of genetic diversity between clonal lines with samples coloured by host plant (**b**) or geographic origin (**c**). **d** Coancestry heatmap of the sampled clones derived from fineSTRUCTURE analysis. The scale shows the degree of shared genetic chunks between the lines (lower, yellow, to higher, blue). The maximum a posteriori (MAP) tree generated by fineSTRUCTURE showing the relationship between samples is shown above the heatmap. The geographic origin of clones and the host plant from which they were collected are indicated by the outer and inner coloured rectangles respectively (see PCA keys for interpretation of colours).

To investigate the ecological and evolutionary factors influencing the emergence and spread of insecticide resistance in *M. persicae*, we first explored population structure in our dataset. A maximum likelihood (ML) phylogenetic analysis of more than 1 million neutrally evolving SNPs recovered highly supported monophyletic clades (bootstrap values of >95%), structured by host plant association, and, to a lesser extent, geographic location (Fig. 2a, and Supplementary Fig. 1). Almost all (26/27) clones from tobacco were contained in a single clade, despite their diverse geographic origin (5 countries and 4 continents). This supports the hypothesis that a single ancestral lineage successfully established and diversified on this host^12,27^. Significant host-associated clustering was also apparent for clones from peach/nectarine (i.e. *Prunus persica*), with 46/51 clones occurring in the *P. persica* dominated clades. Loose groupings of clones from other host species were also observed. For example, 5 out of 10 clones from pepper grouped in a single highly supported clade, while 6 out of 11 clones from oilseed rape grouped with a single clone from broccoli and a single clone from tomato, and a further 3 oilseed rape clones formed an exclusive grouping. Nested within host plant groupings, clones also frequently grouped in subclades on the basis of country of origin. Further investigation of the phylogenetic relationships between the sequenced clones by neighbour-net network analysis supported the topology of the ML tree, and the clustering of clones on the basis of host plant and geography, while displaying evidence of reticulate evolution among deeper splits, consistent with the lower clade support values observed for more basal nodes in the ML phylogeny (Supplementary Fig. 2).

**Fig. 3:**
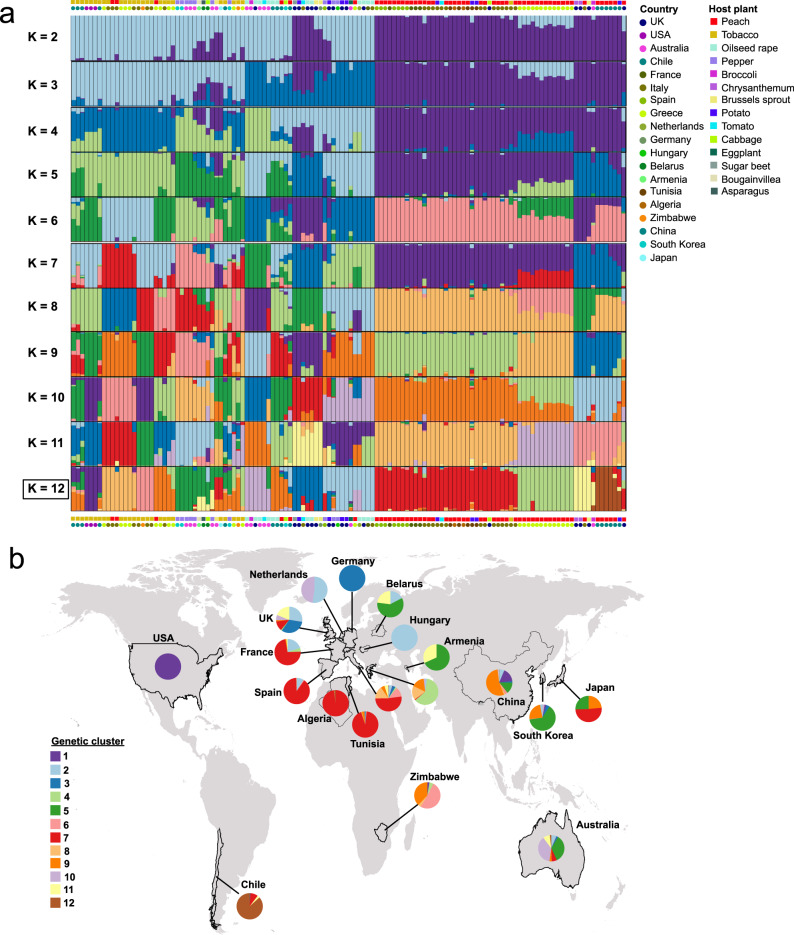
Genetic structure in globally sampled *M. persicae*. **a** Admixture analysis of genetic structure and individual ancestry. Colours in each column represent the inferred proportion of ancestry when K is varied from 2 to 12, the most likely number of predicted genetic clusters (K = 12) is indicated by a box. The geographic origin of clones and the host plant from which they were collected are indicated above and below the structure plot by coloured circles and squares respectively. **b** Geographic representation of genetic structure in the clones when grouped by country of origin (K = 12).

PCA analyses reinforced the observed phylogenetic patterns, with clones clustering by certain host plants (tobacco and peach) and geographic location (Figs. 2b, 2c, and Supplementary Fig. 3). ADMIXTURE^28^ analysis partitioned genetic variation into 12 genetic clusters distributed worldwide (i.e., optimal *K* = 12, Supplementary Fig. 4), and again suggested that population structure in global *M. persicae s.l*. is influenced by host plant and geographic location (Fig. 3a). Haplotype-based analysis using fineSTRUCTURE^29^, which provides greater power to detect subtle levels of genetic differentiation^29^, supported two levels of genetic structure, at a higher level differentiation by host plant, and at a finer scale by geographic location (Fig. 2d). Two large clusters broadly encompassed clones from tobacco and peach respectively, however, other clones again exhibited a degree of clustering on the basis of other hosts, such as pepper and oilseed rape. Finer clusters were frequently based on geographic location with a high degree of co-ancestry commonly observed among samples collected from the same country (Fig. 2d). Finally, to formally test the hypothesis that host plant and geography play a significant role in partitioning genetic variation in *M. persicae s.l*., hierarchical analysis of molecular variance (AMOVA) based on pairwise *F*_ST_ values was performed. This analysis confirmed that both host plant and geography are significant factors in structuring *M. persicae s.l*. populations (p = <0.0001 in both cases), explaining 6.3% and 5.3% of the total variation in the data respectively (Supplementary Table 2).

Taken together, our population genomic analyses provide evidence of genetic differentiation in globally sampled *M. persicae s.l*. based on geography and host plant association. However, they also reveal significant admixture and high connectivity between populations. In terms of geography, particularly strong migration and/or gene flow was evident between certain populations in Southern Europe and Northern Africa, and certain populations in Europe and Asia with those in Australia (Fig. 3b). Previous studies have suggested that long-distance migration is uncommon in *M. persicae*^16,30^. Thus, the spread of genotypes over distant geographic areas is likely a result of anthropogenic factors, including long-distance transport and trade, and the globalization of agriculture. Our analyses of the influence of host plant on *M. persicae* s.l. population structure worldwide provide the first whole-genome level support for a tobacco-adapted race/subspecies (i.e. the *M. p. nicotianae* taxon), the legitimacy of which has been previously called into question^31,32^. Our results also imply a degree of genetic differentiation in lineages associated with other host plants, namely peach, pepper and oilseed rape. However, it is important to acknowledge that the patterns we observe for clones derived from these host plants require further investigation due to current limitations in sampling across different host plants and regions. Specifically, with the exception of *M. persicae* clones from tobacco and peach, sample sizes for clones derived from other host plants in this study are small (in all cases *n* < 11) (Supplementary Data 1). Thus, further more extensive sequencing of clones from other hosts is required to determine the precise status and inclusivity of these groupings.

**Fig. 4:**
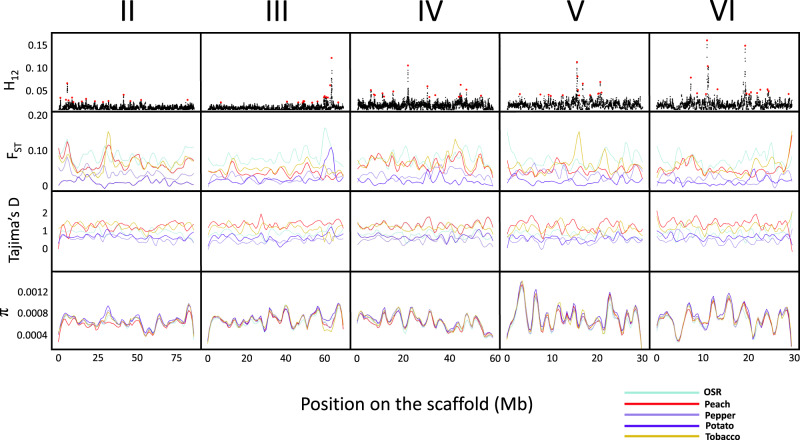
Genomic divergence and signatures of selection associated with host plant use in *M. persicae*. Panels from bottom to top display nucleotide diversity (π), Tajima’s D, F_ST,_ and H12 values across the 5 autosomal chromosomes of *M. persicae* for the main host plant groups (oilseed rape (OSR), peach, tobacco, pepper and potato), see Supplementary Data 1 for sample sizes. H12 scan: Each data point represents the H12 value calculated based on a 1000 SNP window. Red points highlight the top 15 peaks at each scaffold. Fixation index (F_ST_), Tajima’s D, and nucleotide diversity (π): smoothed lines were estimated based on a 10 kb chromosomal window.

To explore the genomic landscape of divergence among putative host-associated populations of *M. persicae*, and identify candidate genomic regions exhibiting signatures of selection associated with host–plant use, we calculated *F*_*ST*_, Tajima’s D and nucleotide diversity (π) statistics in windows of 10 kb across the five autosomes, and scanned for hard and soft selective sweeps using the homozygosity statistic H12 using a 1000 SNP sliding window^33^. This analysis revealed a heterogeneous pattern of divergence, with interspersed peaks and valleys (Fig. 4), consistent with the results of genome scans conducted on host-associated populations of several other insect species^34^. H12 analysis revealed several peaks across all five autosomes as candidate targets of selection differentiating host lineages, with high *F*_*ST*_ values and reduced Tajima’s D and nucleotide diversity in the majority of these regions providing additional evidence that these loci may be involved in adaptive divergence (Fig. 4). Curation of genes residing in 15 of these peaks per autosome identified genes encoding a range of biological functions, including olfactory recognition, digestion, detoxification and excretion, and nucleic acid binding (Supplementary Data 4). The function of these genes suggests roles in host plant recognition and exploitation; however, further functional analyses are required to test this hypothesis. It is also important to acknowledge that genetic differentiation between insect host-plant lineages at specific loci can result from a range of extrinsic and/or intrinsic factors that may be functionally unrelated to host–plant specialization^35^. For example, in aphids genetic differentiation associated with host use may also be influenced by variation in reproductive mode. Specifically, *M. persicae* clones from peach represent cyclical parthenogens (CP) that reproduce sexually once a year. In contrast, clones from field crops may be CP, obligate parthenogens (OP) that reproduce asexually all year round, or functional parthenogens (FP) exhibiting a range of variation in their ability to produce sexual morphs^36,37^. This variation can result in genetic divergence between CP and OP/FP clones as a consequence of reproductive isolation, and potentially in genes controlling reproduction. Thus, future experimental validation of the reproductive capacity of the clones sampled from field crops in this study is required to explore the influence of reproductive mode on observed host-associated genetic divergence.

### Insecticide resistance mechanism in global populations of *M. persicae*

The subdivision of a single insect species into populations that specialize on specific host plants (such as tobacco-adapted *M. persicae*) can lead to partial reproductive isolation between host races and thus reduced gene flow. In the case of insect crop pests, this can have significant applied implications. For example, barriers limiting genetic exchange can strongly affect the emergence and spread of genes conferring resistance to insecticides. To explore the evolution of insecticide resistance in worldwide samples of *M. persicae*, and the extent to which host-plant associations have influenced its development, we first interrogated our genomic data for known resistance mutations. Specifically, we examined the following five known mechanisms: (a) voltage-gated sodium channel (VGSC) knock-down resistance (*kdr*) mutations L1014F, M918T, M918L, that lead to pyrethroid resistance^38–41^; (b) the acetylcholinesterase enzyme mutation S431F, conferring resistance to dimethylcarbamates^42,43^; (c) the γ-aminobutyric acid (GABA) receptor resistant to dieldrin (*Rdl*) mutation A302G, conferring resistance to cylodiene insecticides^44^; (d) the nicotinic acetylcholine receptor mutation R81T, conferring high-level resistance to neonicotinoids^45^; and, (e) mutations leading to amplification of the *CYP6CY3* gene, which confers moderate levels of resistance to neonicotinoids^19,46^.

**Fig. 5:**
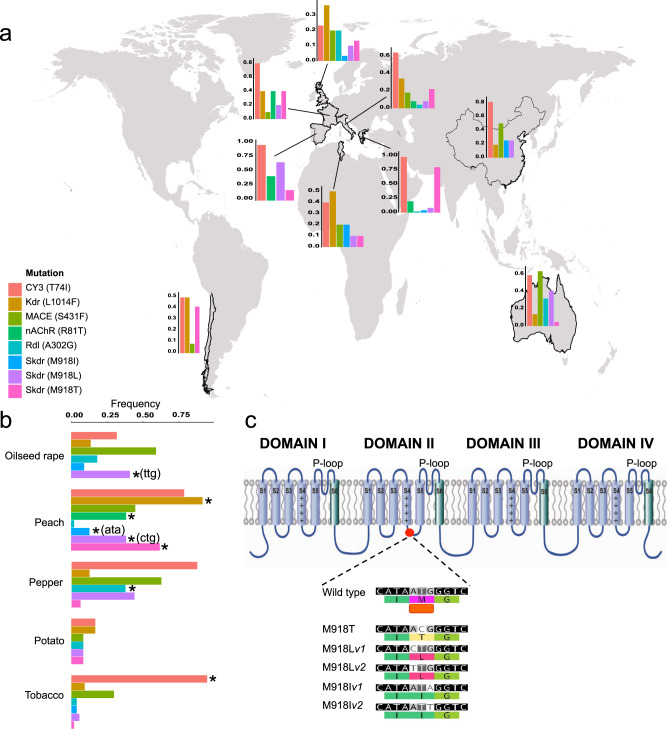
Insecticide resistance mechanisms in global *M. persicae*. **a**, **b** Frequency of eight resistance mutations in *M. persicae* collected from different countries (**a**) and host plants (**b**). Significant (*p* < 0.05) associations between specific resistance mutations and host-differentiated populations are denoted using a star (Fisher’s exact test). Significance applying to a specific codon is indicated in brackets. See Supplementary Data 1 for sample sizes. **c** Identification of novel resistance mutations in domain II of the voltage-gated sodium channel (VGSC) in *M. persicae* that confer resistance to pyrethroid insecticides. A schematic of the VGSC is shown above a nucleotide alignment illustrating the nature and position of two new mutations that both result in the same M918I substitution. For reference the wildtype sequence and the mutations leading to the amino acid substitutions reported previously at this position in resistant *M. persicae* are also displayed in the alignment.

Most resistance mechanisms were found to be globally distributed with the exception of the recently emerged mutation R81T, which was only observed in clones from France, Italy, Greece and Spain (Fig. 5a, Supplementary Data 1), and the M918L mutation encoded by the codon ctg^47^, which was restricted to the western Mediterranean basin (Tunisia, Spain, France and Italy). For certain mutations, such as S431F, no strong association by host plant was observed (Fig. 5b, Supplementary Data 1). However, in several other cases we observed significant patterns of association between resistance genes and specific host-differentiated populations as outlined below.

### Mechanisms of resistance to neonicotinoid insecticides and widespread co-option of a host–plant adaptation

The tobacco-adapted lineage, *M. p. nicotianae*, exhibits resistance to nicotine, the natural insecticide produced by tobacco^19^. Using a transcriptomic-led approach we have previously implicated the amplification of the cytochrome P450 gene *CYP6CY*3 in resistance to this allelochemical^46^. However, the number of origins of this mechanism and the extent to which it occurs in *M. persicae* on tobacco or other host plants remains unclear. Interrogation of our dataset revealed that *CYP6CY3* amplification is ubiquitous in clones collected from tobacco (Fig. 5b, Supplementary Data 1). This finding provides additional evidence of the importance of this mechanism in allowing *M. persicae* to utilise this host plant. However, we also observed *CYP6CY3* amplification at high frequency in clones derived from other host plants (Fig. 5b, Supplementary Data 1). The presence of this mechanism in clones from non-tobacco hosts likely results from the fitness benefits it provides in the presence of neonicotinoid insecticides^19^. We have previously demonstrated that *CYP6CY3* is tandemly duplicated in *M. p. nicotianae* as a large amplicon of ~325 kb creating characteristic breakpoints identifying the region^46^. To investigate the number of evolutionary origins of this mutation, we searched for the presence of these conserved markers in the sequenced dataset and observed a perfect association of the exact breakpoint with the presence of *CYP6CY3* amplification in all clones (Supplementary Data 1). The finding that the mechanism of *CYP6CY3* amplification is identical in all clones, regardless of geographical origin, strongly supports a single origin of *CYP6CY3* amplification in *M. p. nicotianae*, that subsequently spread into *M. persicae s.s*. around the world following the introduction of neonicotinoids. In further support of this, while all clones from tobacco, even those collected prior to the introduction of neonicotinoids in 1991, have amplified *CYP6CY3*, the 6 clones in the dataset from non-tobacco hosts that were collected prior to 1991 lack this mechanism (Supplementary Data 1). Thus, our data suggest that a pre‐existing host adaptation has been co‐opted as a resistance mechanism, and neonicotinoid resistance, previously a co‐incidental pleiotropic effect, has become the major selective force driving the geographic expansion of this trait.

More recently, an additional mechanism of resistance to neonicotinoid insecticides has emerged in *M. persicae* resulting from an amino acid substitution, R81T, in the target-site of this insecticide class, the nicotinic acetylcholine receptor^45^. We find this mechanism to be significantly associated with clones derived from peach (Fisher’s exact test *p* = <0.001, Fig. 5b, Supplementary Data 1), and haplotype analysis suggested this mechanism has a single origin, consistent with its recent, localised emergence (Supplementary Fig. 5). Intriguingly, this mutation was only observed in clones displaying *CYP6CY3* amplification (Supplementary Data 1). This suggests that R81T emerged on a genetic background of *CYP6CY3* overexpression, and that the association has been maintained, despite the fact that the two mutations occur on different chromosomes. The continued selection for clones with both mechanisms suggest they provide strong fitness benefits in combination, consistent with previous work suggesting the mechanisms may act in synergy to confer high levels of resistance to neonicotinoids^45^. Thus, the evolution of resistance to neonicotinoids in *M. persicae* likely represents an adaptive walk with a metabolic mechanism originating from host-plant adaptation, first co-opted to confer moderate resistance, with subsequent evolution of target-site resistance acting in concert to confer potent insecticide resistance. Notably, and in contrast to the distribution of *CYP6CY3* amplification, the pattern of R81T prevalence observed in Europe suggests barriers to gene flow between aphids on tobacco and those on peach. Specifically, while clones collected from peach in several countries carry this mechanism, sympatric populations from tobacco do not, even when collected from the same vicinity and at the same time (Supplementary Data 1). Thus, while the distribution of *CYP6CY3* amplification suggests that given sufficient time, in the face of strong selection, alleles conferring strong fitness benefits can spread between host-associated populations, the distribution of R81T suggests that barriers to gene flow between specific host-associated populations can slow the rate of transfer of such alleles.

### Mechanisms of resistance to pyrethroid insecticides—multiple origins and novel mutations

Resistance to the widely used pyrethroid insecticides in *M. persicae* is conferred by amino acid substitutions in the voltage gated sodium channel, with three amino acid substitutions previously described in resistant populations: (i) the *kdr* mutation L1014F, and the two *super-kdr* (*skdr*) variants, (ii) M981T, and (iii) M918L^38–41^. In the case of M918L, two different forms have been described encoded by the codons ctg or ttg^41,47^. In the current study, all three amino acid substitutions were observed at varying frequencies and in multiple combinations in populations across the sampled range, with the exception of M981L_ctg (see above) (Fig. 5a, Supplementary Data 1). Contrasting patterns were observed in the distribution of the two *skdr* variants. M918T, which was described first^2^, was significantly associated with clones from peach (Fischer’s exact test, *p* = <0.001), where it was found at high frequency. In contrast, this mutation was observed at only very low frequency in pepper and tobacco, and was not found in any clones from oilseed rape, where the alternative *skdr* variant M918L (encoded by ttg) was common. While our sample size of clones derived from oilseed rape is small, this finding mirrors that of previous studies of *M. persicae* from this host plant, which also failed to identify M918T in populations on oilseed rape in France^41,48^. Thus, further sequencing of sympatric *M. persicae* populations from peach and OSR is warranted, to confirm if barriers to resistance gene flow exist between populations on these host plants, and if so how these operate.

In addition to the previously described *kdr*/*skdr* mutations, our analyses also uncovered a novel mutation in the VGSC at amino acid position 918. This results in the replacement of the wild-type methionine at this position with isoleucine (Fig. 5c). All clones with this mutation carried it in the heterozygous state, in combination with either the wildtype allele M918, or one of the two other *skdr* alleles L918 and T918^40,41^. Furthermore, the M918I mutation was observed in clones both with and without the *kdr* L1014F mutation (Supplementary Data 1). While the M918I substitution has not been previously reported in *M. persicae* it has been described in other pyrethroid resistant strains of insects^49,50^. In addition, VGSC isoforms of mammals, which exhibit low sensitivity to pyrethroids, encode isoleucine at the equivalent position, and substitution of isoleucine for methionine at this position in the rat IIA α‐subunit causes a 100‐fold increase in sensitivity to pyrethroids^51^. This both demonstrates the causal role of M918I in resistance and suggests the isoleucine at this position in the VGSC of mammals, at least in part, explains its low sensitivity to pyrethroids^51^. Thus, in this instance, *M. persicae* has evolved resistance by becoming more ‘mammalian-like’, overcoming, at least in part, the insect-specificity of this insecticide class.

The discovery of the novel M918I mutation means that a total of three independent amino acid substitutions have arisen at the same position in *M. persicae*, each conferring nerve insensitivity to pyrethroid insecticides. The repeated evolution of distinct *skdr* mutations in *M. persicae* is thus an excellent demonstration of the remarkable evolvability of this species. It also illustrates how strong and continuous selection pressure imposed from insecticide use can lead to a diversity in the ‘evolutionary solutions’ to the same environmental challenge, even in highly conserved and functionally constrained, insecticide target proteins.

Remarkably, two alternative codons encoding isoleucine were observed in the 13 clones carrying M918I (5 clones with the codon ATT and 8 clones with the codon ATA) (Fig. 5c, Supplementary Data 1), strongly suggesting that the same amino acid substitution has independently evolved on at least two occasions. Thus, while haplotype analysis failed to resolve the precise number of times each unique kdr and skdr mutation observed in the sampled clones have emerged (Supplementary Fig. 6), the discovery of five different mutations at the M918 locus indicates that skdr resistance has independently evolved at least five times in global populations of *M. persicae*.

### Population genomics of variation in sensitivity to a recently introduced insecticide

**Fig. 6:**
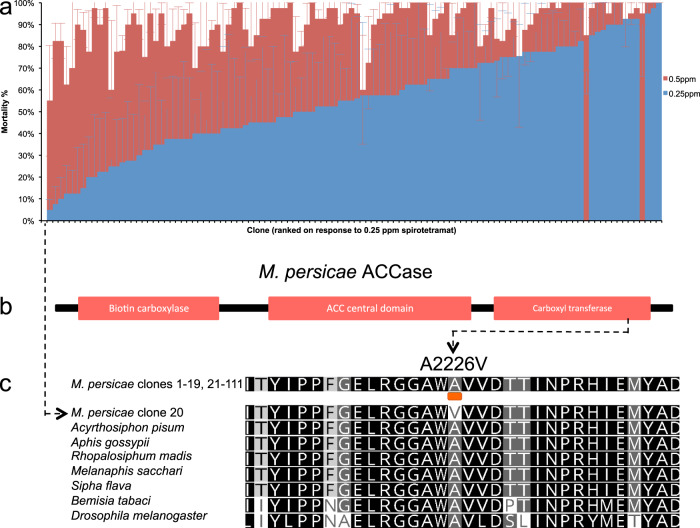
Population genomics of variation in sensitivity to a recently introduced insecticide. **a** Sensitivity of 110 clones of *M. persicae* to two concentrations (0.25 ppm and 0.5 ppm) of the insecticide spirotetramat. Error bars display 95% confidence intervals (*n* = 4 biological replicates, each comprising 10 aphids). **b**, **c** Identification of a novel resistance mutation in a highly conserved region of the acetyl-CoA carboxylase (ACC) enzyme carboxyltransferase (CT) domain in *M. persicae* that confers resistance to spirotetratmat. A schematic of the ACC enzyme is shown above an amino acid alignment illustrating the position of an alanine to valine substitution in clone 20 (that exhibits marked resistance to spirotetramat) that was not observed in any of the other *M. persicae* clones. To illustrate the conserved nature of the alanine at this position across insects the sequences of several other insect species are included in the alignment.

Because of the widespread resistance in *M. persicae* to older classes of insecticide, growers are increasingly reliant on just a handful of newer modes of action for control. One such compound is spirotetramat, which belongs to the tetronic/tetramic acid (cyclic ketoenol) family. The efficacy of this insecticide is not compromised by pre-existing resistance to older insecticide classes and, to date, no examples of *M. persicae* with resistance to spirotetramat have been described^52,53^. To explore the utility of the living clone library and matched genomic resources developed in this study for genotype-phenotype mapping we examined the response of 110 *M. persicae* clones to two concentrations of this insecticide (0.25 ppm and 0.5 ppm) (Fig. 6a). For 109 of the clones a gradient in response to treatment with 0.25 ppm spirotetramat was observed ranging from 5% to 100% mortality. This clearly demonstrates the considerable phenotypic variation in sensitivity to low concentrations of this insecticide among clones. However, this variation in tolerance is unlikely to impact on control in the field, as when treated with 0.5 ppm spirotetramat mean mortality across the tested clones was >90%, with no clones exhibiting mortality <55%. In contrast, a single clone collected in Queensland, Australia exhibited a marked difference in its response to spirotetramat, and, remarkably, was unaffected (0% observed mortality) by either concentration of this insecticide.

The identification of a single resistant clone provided insufficient power for genome-wide association analyses (GWAS). Thus, to investigate the mechanistic basis of resistance in this clone we interrogated sequence reads mapped to the gene encoding the target of spirotetramat, the acetyl-CoA carboxylase enzyme (ACC, EC 6.4.1.2) (Fig. 6b). This analysis revealed a single non-synonymous mutation (gCt > gTt) in the heterozygous state at position 2,226 in the Queensland clone that was not observed in any of the other sequenced clones. This mutation results in an alanine to valine substitution and occurs at an amino acid residue in a highly conserved region of the ACC carboxyltransferase (CT) domain (Fig. 6b, c). Significantly, while previously undescribed in aphids, the same amino acid substitution at the equivalent position has very recently been described in spirotetramat resistant strains of the whitefly *Bemisia tabaci*^54^ and the model nematode *C. elegans*^55^, demonstrating repeated evolution of the same resistance mechanism across phylogeny. Furthermore, the creation of *Drosophila melanogaster* lines with the alanine to valine mutation in the orthologous ACC gene by CRISPR-Cas genome editing has shown that this mutation confers potent (>800-fold) resistance to spirotetramat, and strong cross-resistance to other ketoenols^54^. Together, these studies provide unequivocal evidence of the causal role of this mutation in resistance to ketoenols. The discovery of this mutation means that at least eight independent mechanisms of resistance to insecticides belonging to six different classes have now been identified in *M. persicae*. Notably, the majority of these mechanisms have been shown to involve single genes/mutations of large effect, rather than multiple genes of minor effect. This finding is consistent with theory, which suggests that selection for phenotypes outside the normal phenotypic distribution favours a monogenic response, i.e. a rare allele at a single locus that can confer substantial resistance immediately^56,57^.

The discovery of resistance to spirotetramat in *M. persicae* is concerning as this compound remains one of just a handful of insecticide modes of action that have, to date, not been compromised by resistance. Fortunately, the lack of the mutation in any of the other sequenced clones, including 10 from Australia, suggests we have detected resistance at an early stage. This, in combination with characterisation of the underpinning mechanism of resistance, will facilitate the development of diagnostic assays to monitor for spirotetramat resistance in global populations of *M. persicae*. Such information is a prerequisite for the development and deployment of strategies to manage the spread of resistance and preserve the life of this important insecticide.

### Linkage disequilibrium

**Fig. 7:**
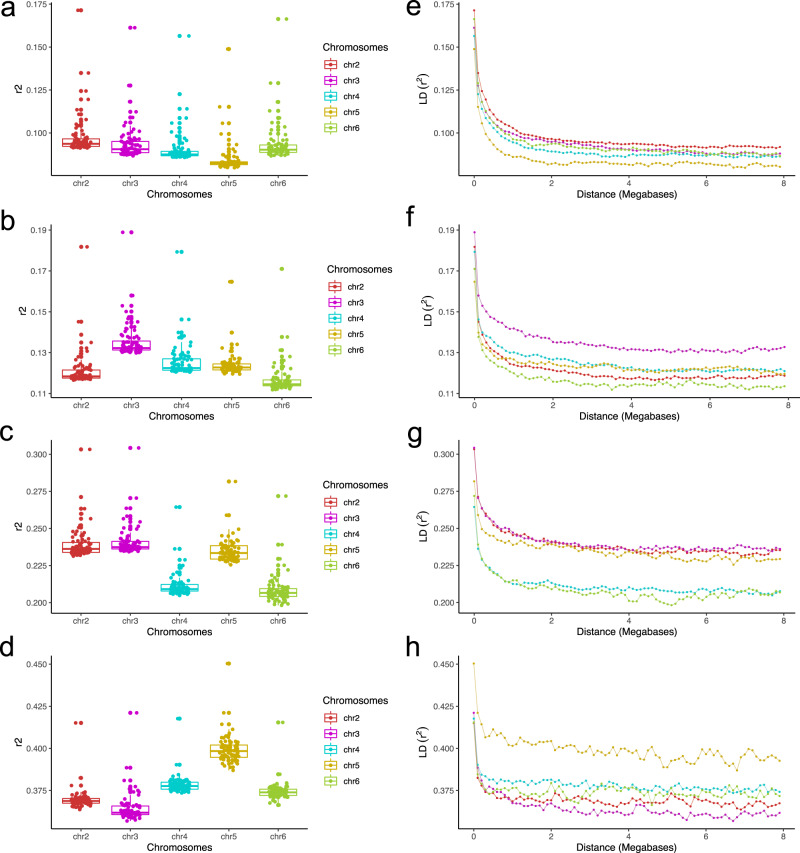
Average long-range linkage disequilibrium (LD) and LD decay over distance for all autosomes of *M. persicae* from peach and tobacco in Italy and Greece. Distribution of r^2^ is plotted separately for all autosomes in clones from **a** peach-Greece, **b** peach-Italy, **c** tobacco-Greece and **d** tobacco-Italy. See Supplementary Data 1 for sample sizes. Individual points in the box plot represent mean r^2^ values in 100 KB windows along the entire length of autosomes. r^2^ values are plotted as a function of distance (LD decay) across all autosomes in **e** peach-Greece, **f** peach-Italy, **g** tobacco-Greece and **h** tobacco-Italy clones.

The characterisation of resistance to spirotetramat described above demonstrates the utility of the *M. persicae* clone library and matched genomic resources for phenotype-genotype association using a candidate gene approach. However, in the absence of a priori candidates, the power of GWAS to accurately detect causal variants is strongly influenced by population structure (see above) and linkage disequilibrium (LD)^58^. To inform future GWAS we examined the extent of LD in clones collected from peach and tobacco in Italy and Greece (where our sample sizes are largest) at two scales: the level of the autosome, and at three insecticide resistance gene loci (sites of the *kdr*+*skdr*, S431F, and R81T mutations). Long range LD analysis across all autosomes revealed low to moderate levels of average LD in the populations (mean *r*^2^ values of 0.08–0.39) (Fig. 7a–d), consistent with the levels of LD reported for other insects^59–61^. LD decayed rapidly with distance achieving background levels within 11.5 kb on average (Fig. 7e-h). Consistent with this, the average length of LD blocks for the different autosomes/populations was short, ranging from 6.7 to 16.3 kb (Supplementary Table 3). The levels of long range LD varied with chromosome, and to a greater extent by population, (Fig. 7) with LD higher in populations from tobacco, especially from clones from tobacco in Italy, compared to the two populations from peach. This finding likely reflects differences in the frequency of sexual reproduction in these populations^37,62^, and thus the capacity for recombination to reduce LD. In addition, the differences in LD in populations from peach and tobacco in Italy could be explained, at least in part, by the demographic history of these populations. While further analyses are required to investigate this in detail, the distribution of allele frequencies across polymorphic sites of each autosome, summarised as the site frequency spectrum (SFS), displayed a similar profile in the populations from peach and tobacco, characterized by a majority of low frequency variants (Supplementary Fig. 7). This provides initial evidence that the differences in LD in the populations are not explained by marked changes in the size of the two populations over time.

Across the three resistance loci, mean r^2^ values ranged from 0.2 to 0.5 in clones from peach, whereas in clones from tobacco mean r^2^ values were around 0.1. The highest mean r^2^ values were observed at the R81T loci in clones from peach (0.5), and this correlates with the fact that the R81T mutation is observed in clones from peach but not tobacco. However, as revealed by the grid plot (Supplementary Fig. 8c), the R81T mutation is not positioned in any haploblock suggesting other associated polymorphisms are responsible for the high mean r^2^ value across this loci. Similarly, the MACE and *kdr*/*skdr* mutations do not occur in any significant haploblock, suggesting any association between these resistance mutations and flanking polymorphisms has been broken down by recombination.

In summary, the differences in LD in clones from different host plants/countries will need to be considered in future association analyses. However, the generally low levels of long-range LD observed in our analyses are favourable for GWAS as they facilitate the precision with which causal variants associated with a phenotype of interest can be identified.

## Conclusions

The chromosome-scale assembly, resequenced genomes, and living library of more than 110 *M. persicae* clones generated in this study represents a powerful resource for further research on aphids. Future use of this resource should consider the genetic background of the sampled clones, which, as a global collection, encompasses high phenotypic and genetic heterogeneity. While capturing more variation, this reduces mapping power relative to a collection of individuals from a single sexual population^58^. Nevertheless, we envisage the sequenced panel has strong potential to provide a range of insights into the evolution and genetic basis of many of the remarkable biological traits exhibited by aphids. In the current study we have leveraged this resource to advance understanding of the evolution of insecticide resistance in an important insect pest at a global scale, uncovering both mechanisms underpinning resistance and ecological factors that influence its emergence and spread.

Our data reveal that global populations of *M. persicae* s.l. exhibit evidence of genetic differentiation on the basis of geography and host-plant association. The subdivision of a single insect species into populations that specialize on different hosts, while maintaining an appreciable rate of gene flow, (i.e. host races or biotypes) can have a range of evolutionary and applied implications^63^. Thus, the first whole genome-level support for a tobacco-adapted subspecies in *M. persicae s*.l. provided in this study is significant. The genetic divergence of *M. persicae* on other host plants implied by our analyses also warrants further investigation, including additional sequencing of sympatric populations of *M. persicae* from peach and non-tobacco secondary host plants.

Our investigation of the extent to which host-associated populations in *M. persicae* have influenced the development of insecticide resistance provides several examples that the strong selection pressure exerted by insecticide use can overcome any constraints to gene flow resulting from host plant specialization. This has resulted in the pervasive presence of many resistance mechanisms in this species worldwide. However, we also uncover cases where the evolution and spread of resistance appears to have been influenced by barriers to gene flow between certain host-associated populations, a finding that has implications for resistance risk assessment and management^4^. Investigation of the relationship between host-plant association and insecticide resistance in *M. persicae* has also provided insight into fundamental questions concerning adaptation to novel selective pressures and the origins of novel traits. Specifically, our findings demonstrate that adaptations enabling insect host range expansion can provide a source of genetic novelty than can be rapidly co-opted to provide widespread resistance against synthetic insecticides.

Analysis of the molecular basis of insecticide resistance in this study revealed repeated evolution of novel mutations at the same resistance loci, and uncovered novel mechanisms against key insecticides. The repeatability of evolution is a long-standing fundamental question in evolutionary biology, and, in the context of resistance, also has important practical implications^4^. Our characterisation of mutations that confer resistance to pyrethroid insecticides reveals surprising intraspecific diversity in the evolutionary response of a global insect pest to insecticide selection. The reliability of molecular diagnostics used to inform resistance management depends on whether different populations have evolved the same or different resistance mechanisms. In this regard our findings demonstrate that *de novo* resistance mutations arising in pest populations may show low repeatability, and thus highlight the importance of regularly sampling diverse pest populations for resistance mechanisms, even after resistance has emerged.

In the battle against resistance, detecting the mechanisms that compromise control at an early stage is critical, as it allows interventions to be introduced that limit the spread of resistance mechanisms before they become fixed in a population. Our characterisation of resistance to the recently introduced insecticide spirotetramat, and identification of the causal mutation involved, demonstrate the power of population genomic interrogations to detect resistance at an early stage. Such knowledge is vital if we are to prolong the life of current and future insecticides in order to sustainably control highly damaging global insect pests.

## Materials and methods

### Aphid clones

Full information on the 127 *M. persicae* s.l clones used in this study is provided in Supplementary Data 1. These include both defined subspecies of *M. persicae* s.l., i.e. *M. persicae* s.s. (clones that are not adapted to tobacco) and *M. p. nicotianae* (the tobacco-adapted subspecies). Aphids were collected opportunistically by the authors of this manuscript and their collaborators, and are derived from 14 host plants (primarily from agriculturally important crops) in 19 countries (Fig. 1b, Supplementary Data 1). Of these, 110 are continuously maintained in the Bass laboratory as asexual lineages on individual Chinese cabbage leaves (*Brassica napus* L var *chinensis* cv Tip-Top) in small plastic cups maintained at 18 °C under a 16:8 h light:dark regime. These are available to other researchers as live cultures or preserved material upon request from the corresponding author.

### Sequencing and de novo assembly of the *M. persicae* clone G006

A draft genome of the *M. persicae* clone G006 was previously assembled using Illumina short-read sequencing^8^. Aphids derived from the same asexually reproducing colony were used as a source to improve the genome assembly of this clone here, using PacBio single-molecule real-time (SMRT) sequencing and in vivo chromatin conformation capture (HiC). DNA was extracted from pools of adult aphids using the Genomic-tip kit (Qiagen) according to the manufacturer’s instructions, and used to generate long-read PacBio libraries sheared to a target length of approximately 60 kb (following a 30 kb+ protocol). Libraries were sequenced using five PacBio Sequel SMRT cells, with ~8.5 gb obtained per SMRT cell totalling nearly 40 gb. The publicly available Illumina short-reads for clone G006^8^ were also downloaded from the NCBI short read archive and used for assembly polishing and quality assessment. Short read data were trimmed using TrimGalore -v 0.4.0^64^ with the default settings.

For assembly, we trialled several long-read sequence assemblers including Canu^65^, wtdbg2^66^, Flye^67^ and Falcon^68^. We also investigated the results of merging the output of these assemblies using quickmerge^69^, aiming to maximise genome completeness and minimise duplicated regions caused by under-collapsed heterozygosity. The best results were obtained using error corrected PacBio reads derived from Canu -v1.8.0 to assemble with wtdbg2 -v1.0 and Flye -v2.6.0, with the two assemblies then merged with quickmerge -v0.3. To assess contiguity and gene completeness in the test and final assemblies we used KAT -v1.0.0^70^, and BUSCO –v4.14^22^ applying the Arthropoda gene set (*n* = 1,066). Assemblies were polished iteratively, after every assembly step, using three rounds of Racon -v1.3.1^71^ using the long-read data and 3 rounds of Pilon -v1.22^72^ using the short-read data in diploid mode. Redundant haplotigs were removed in Purge_haplotigs -v1.0.4^73^.

To scaffold the long-read assembly to chromosomal level, Dovetail HiC libraries were prepared as described previously^74^. Briefly, for each library, chromatin was fixed in situ with formaldehyde in the nucleus and extracted. Fixed chromatin was digested with DpnII, the 5′ overhangs filled in with biotinylated nucleotides, and then free blunt ends ligated. After ligation, crosslinks were reversed and DNA purified from the proteins. Purified DNA was treated to remove biotin that was not internal to ligated fragments. The DNA was then sheared to ~350 bp mean fragment size and sequencing libraries were generated using NEBNext Ultra enzymes and Illumina-compatible adapters. Biotin-containing fragments were isolated using streptavidin beads before PCR enrichment of each library. The libraries were sequenced on an Illumina HiSeqX to produce 97 million 2 × 150 bp paired-end reads, which provided 13,068.91X physical coverage of the genome (10–10,000 kb pairs).

The long-read *de novo* assembly and Dovetail HiC library reads were used as input for the Juicer pipeline^75^ to identify HiC contacts. The 3D-DNA assembly pipeline^76^ was then used to first correct mis-assemblies in each input assembly and then to order contigs into super-scaffolds. As K-mer analysis showed that our draft assemblies did not contain substantial quantities of duplicated content caused by the inclusion of haplotigs, the 3D-DNA pipeline was run in “haploid mode” and with an—*editor-repeat-coverage* of 4. The initial HiC assemblies were then manually reviewed using Juicebox Assembly Tools (JBAT) to correct mis-joins and other errors^77^. Following JBAT review, the assemblies were polished with the 3D-DNA seal module to reintegrate genomic content removed from super-scaffolds by false positive manual edits, to create a final scaffolded assembly. The HiC assemblies were then screened for contamination with BlobTools^78^. Finally, a frozen release was generated with scaffolds renamed and ordered by size with SeqKit v0.9.1^79^. The final assemblies were checked with BUSCO and KAT comp to ensure the scaffolding and decontamination steps had not reduced gene-level completeness or removed genuine single-copy aphid genome content.

### Annotation of the G006 assembly

Prior to gene prediction the assembly was soft masked for repetitive elements with RepeatMasker -v4.0.7^80^ using repeat libraries generated by RepeatModeler -v2.0.2 [https://github.com/Dfam-consortium/RepeatModeler]. Protein coding genes were predicted using GeneMark-ES –v4.3.8^81^ and AUGUSTUS –v3.3.0^82^ implemented in the BRAKER -v2.1.2^83^ pipeline using publicly available RNA-seq datasets^8,46^ as evidence. RNA-seq datasets were mapped against the repeat masked genome using HISAT2 v2.0.5^84^ with the parameters—*max-intronlen* 25000 *–dtacufflinks* &*—rna-strandness* RF followed by sorting and indexing with SAMtools -v1.3^85^. BRAKER2 was run with UTR training and prediction enabled with the parameters *–softmasking*—*gff3*—*UTR* = on. Strand-specific RNA-seq alignments were split by forward and reverse strands and passed to BRAKER2 as separate BAM files to improve the accuracy of UTR models as recommended in the BRAKER2 documentation. Following gene prediction, genes that contained in frame stop codons were removed using the BRAKER2 script getAnnoFastaFromJoingenes.py and the completeness of each gene set was checked by BUSCO analysis using the longest transcript of each gene as the representative transcript. Functional annotation of the *de-novo* predicted gene models was performed based on homology searches against the NCBI nr and Interpro databases using BLAST2GO –v5.2.5.

### Population sample resequencing and variant calling

Sequence data for 17 of the clones utilised in this study has been described previously^46,86^. For the remaining clones DNA was extracted from pools of 10-20 aphids of each clone using the E.Z.N.A.® Insect DNA Kit (Omega Bio-tek) and used to construct PCR-free libraries. Libraries were sequenced on a NovaSeq6000 using a 150 bp paired-end read metric to an average coverage of 40X. FastQC was used to check the quality of the raw reads obtained^87^ and reads were trimmed using TrimGalore^64^. For species validation sequence data for all clones was aligned to the Cytochrome C oxidase subunit gene derived from the most recently published *M. persicae* mtDNA genome^88^ using Geneious (Biomatters), and alignments of each clone were manually inspected. To call variants, data were first aligned to the chromosome-scale assembly of clone G006 assembly using BWA -v 0.7.17^89^. PCR duplicate reads were removed from alignments and the remaining data were sorted using SAMtools -v 1.9.0^85^. Variants were called using the genome analysis toolkit GATK -v 4.1.0^90^ haplotypecaller function. Individual genomic VCF records (gVCF) were jointly genotyped using GATK’s genotype GVCFs. Genotype calls were filtered for minimum depth (DP) of ≥ 10. Variant calls with a minimum genotype quality (GQ) ≥ 30 were further retained. 45,627,645 high quality allelic variants were retained after variant calling and filtering of low-quality calls. We used 0.01 as the minor allele frequency cut-off. The final alignment had 1,064,888 columns and 130 rows with 105,979 distinct patterns. Among these, 1,017,412 sites were parsimony-informative, 33,660 were singleton sites and 13,816 constant sites. The specific data filtering steps prior to running population structure and phylogenetic analyses are explained in the respective sections. Detailed description of the workflow used for the analysis of the population genomic data in this study is available as a Jupyter Notebook on GitHub https://github.com/cordeiroemg/Myzus_PopGen_Workflow.

### Analysis of obligate and facultative symbionts

To explore the occurrence and distribution of bacterial endosymbionts and other microbes present in the sampled *M. persicae* clones we used a framework recently developed for metagenomic analysis of aphids^24^. Read sets were first mapped using BWA-MEM^89^ to a collection of >30 reference genomes of known aphid symbionts, their associated plasmids, and a number of viruses known to infect aphids^24^. Following this mapping step, several statistics were computed, including mapping rate, average coverage for each genome, fraction of the reference genome covered by at least five reads, and mean edit distance for the reads mapping on each reference genome. Unmapped reads from this analysis were extracted using Samtools^85^, low quality reads were removed using Trimmomatic^91^, and the remaining reads were taxonomically assigned using Kraken2^92^ and Centrifuge^93^ to identify microbial sequences not represented in the collection of reference genomes used in the first round of analysis.

### Pairwise comparisons of genetic distance

The genetic divergence between the sequenced *M. persicae* clones was initially assessed by creating a simple distance matrix of pairwise clone comparisons using the generic *dist()* function in R programming environment -v3.6.1.

### Phylogenetic analyses

The VCF file, with more than 1 million variant sites, was converted to PHYLIP format using a custom python script. The final alignment had 1,064,888 columns and 130 rows with 105,979 distinct patterns. Among these, 1,017,412 sites were parsimony-informative, 33,660 and 13,816 were singleton and constant sites respectively. Phylogeny was estimated using maximum-likelihood (ML) inference in IQTree -v 1.6^94^, using the TVM + F + R5 + ASC substitution model with correction for ascertainment bias and 10,000 traditional bootstrap replicates (-cmax 15 -B 10000 -alrt 10000 -bnni -T AUTO). SplitsTree -v4.16.1^95^ was used to create a distance-based split network using the neighbour-net algorithm.

### Population structure and gene flow

SNPs were filtered in Plink 1.9^96^. Only biallelic SNPs under Hardy-Weinberg Equilibrium using 5% probability were used. Moreover, only SNPs with 0 missing rate and MAF > 0.05% were included in genetic structure analyses. Principal Component Analysis (PCA) was performed in R using the *dudi.pca* function of the *ade4* package^97^. Model-based analyses employed ADMIXTURE^28^ with a range of population sizes explored ranging from K = 1 to K = 20. The most likely number of genetic clusters was determined by the inspection of the cross-validation error, in which the smallest value indicated the best estimate of K. Co-ancestry relationships and fine scale analysis of genetic structure was explored using fineSTRUCTURE^29^. To formally test the hypothesis that host plant and geography play a significant role in partitioning genetic variation in *M. persicae* hierarchical analysis of molecular variance (AMOVA)^98^ was performed using Arlequin v. 3.52^99^. Groups for host plant included peach, tobacco, pepper, oilseed rape, for geographic location Europe, Africa, Asia, Australia, Asia, South America, and North America.

### Selection scans

Divergent regions of the genome between populations from different host plants were identified by calculating pairwise F_ST_ values, nucleotide diversity (*π*), and Tajima’s D using VCFtools version 0.1.14^100^. Average values of F_ST_, *π*, and Tajima’s D were calculated based on 3 million SNPs. F_ST_ values were also calculated for each individual SNP, and *π*, and Tajima’s D for non-overlapping 10-kb windows. We further scanned the genome for signatures of selection by H12 analysis, a haplotype-based approach that uses phased SNPs to detect selection sweeps^33^, selecting the 15 highest peaks on each autosome, and retrieving lists of genes within candidate divergent regions from .*bff* annotation files.

### Sequence analysis of candidate genes

Sequence variation in candidate genes was manually analysed by mapping reads of each clone to regions encompassing these genes using BWA^89^, or the ‘map to reference’ function of Geneious -vR9, with alignments visualised using the Geneious software suite. Significant (p < 0.05) associations between specific insecticide resistance mechanisms and host-differentiated populations of *M. persicae* were identified using Fisher’s exact test performed in R.

### Analysis of linkage disequilibrium and site frequency spectrum

To estimate the decay of LD on individual autosomes for clones from peach and tobacco from Italy and Greece variants were thinned using MapThin -v1.11^101^ and PLINK -v1.90b4^96^ used to estimate inter-variant allele count squared correlations (r^2^). Plots of r^2^ as function of distance were created using custom R scripts. The level of LD at three insecticide resistance loci was examined using HaploView -v02032021^102^. PLINK -v1.90b4^96^ was used to initially convert the variants data into HaploView native format. The site frequency spectrum (SFS) of polymorphisms on different autosomes was estimated using ANGSD -v0.921^103^.

### Haplotype analyses of resistance mutations

Illumina short-reads derived from each *M. persicae* clone were mapped against the reference gene sequences encoding the nicotinic acetylcholine receptor β1 subunit and the voltage-gated sodium channel using BWA-MEM^89^. Consensus sequences were called from the BAM file for each clone using bcftools -v 1.9 *mpileup* and *consensus* utilities^104^, aligned with MAFFT -v7.471^105^ using the *–auto* mode. FASTA alignments then converted to PHYLIP format and used to generate phylogenetic trees using IQTree-v 1.6^94^. Phylogenetic networks were created using the TCS -v1.21 software suite^106^.

### Spirotetramat bioassays

Aphids were age synchronized to generate 3-4 day old nymphs for testing. To determine optimal discriminating doses for testing the entire library of *M. persicae* clones, assays were initially performed on five representative clones using 7 spirotetramat concentrations spanning 0.0488 ppm – 12.5 ppm. This informed the choice of two discriminating doses – 0.25 ppm and 0.5 ppm—for screening all other clones. The sensitivity of each clone to the two spirotetramat concentrations, and a non-insecticide control, was tested in a leaf-dip bioassay using four biological replicates each comprising 10 nymphs. *Brassica rapa* leaf discs 37 mm in size were immersed in the appropriate concentration of insecticide solubilized in acetone and diluted in 0.02% Triton/H2O for 10 s. For controls, leaves were immersed in diluent minus insecticide. Discs were air-dried before being placed abaxial side up on 1% agar in discrete pottles, to which 10 nymphs were added. All bioassays were kept at 24 °C ± 1 with a photoperiod of 16:8, and each assay was scored for mortality at 72 hours. Aphids that were unable to control motor-function (e.g. could not right themselves when flipped) were recorded as ‘affected’ and were included in mortality data.

### Statistics and reproducibility

Statistical analysis of data was performed using R as described above. For all statistical analysis, data from at least three independent measurements was used. The exact number of replicates are indicated in individual figure captions and the methods.

### Reporting summary

Further information on research design is available in the Nature Research Reporting Summary linked to this article.

## Supplementary information

Supplemental Material

Description of Supplementary Files

Supplementary Data 1

Supplementary Data 2

Supplementary Data 3

Supplementary Data 4

Reporting Summary

## Data Availability

The sequence data generated in this study has been deposited with NCBI under the Bio Project ID: PRJNA574571^107^. For individual accession numbers associated with each clone see Supplementary Data 1. The genome assembly of *M. persicae* clone G006 is also available at AphidBase https://bipaa.genouest.org/is/aphidbase/^108^. Source data for figures are available in Supplementary Data 1 and on Dryad 10.5061/dryad.vhhmgqnt7. All other data generated during the current study is available from the corresponding author on reasonable request.

## References

[1] Miles A (2017). Genetic diversity of the African malaria vector *Anopheles gambiae*. Nature.

[2] Bass C (2014). The evolution of insecticide resistance in the peach potato aphid, *Myzus persicae*. Insect Biochem. Mol. Biol..

[3] Zimmer CT (2018). Neofunctionalization of duplicated P450 genes drives the evolution of insecticide resistance in the brown planthopper. Curr. Biol..

[4] Hawkins NJ, Bass C, Dixon A, Neve P (2018). The evolutionary origins of pesticide resistance. Biol. Rev..

[5] Crossley, M., H., C. Y., Groves, R. H. & D., S. S. Landscape genomics of Colorado potato beetle provides evidence of polygenic adaptation to insecticides. *Mol. Ecol*. **26**, 6284–6300 (2017). 10.1111/mec.14339 28857332

[6] van Emden, H. F. & Harrington, R. *Aphids as crop pests*. (CABI, 2017).

[7] Consortium IAG (2010). Genome sequence of the pea aphid *Acyrthosiphon pisum*. Plos Biol..

[8] Mathers, T. C. *et al*. Rapid transcriptional plasticity of duplicated gene clusters enables a clonally reproducing aphid to colonise diverse plant species. *Genome Biol*. **18**, 10.1186/s13059-016-1145-3 (2017). 10.1186/s13059-016-1145-3 PMC5304397 28190401

[9] Mathers TC (2020). Chromosome-scale genome assemblies of aphids reveal extensively rearranged autosomes and long-term conservation of the X chromosome. Mol. Biol. Evol..

[10] Li Y, Park H, Smith TE, Moran NA (2019). Gene family evolution in the pea aphid based on chromosome-level genome assembly. Mol. Biol. Evol..

[11] Chen W (2019). Genome sequence of the corn leaf aphid (*Rhopalosiphum maidis* Fitch). Gigascience.

[12] Margaritopoulos JT, Kasprowicz L, Malloch GL, Fenton B (2009). Tracking the global dispersal of a cosmopolitan insect pest, the peach potato aphid. BMC Ecol..

[13] Peccoud J, Ollivier A, Plantegenest M, Simon JC (2009). A continuum of genetic divergence from sympatric host races to species in the pea aphid complex. Proc. Nat. l Acad. Sci. U. S. A..

[14] van Emden, H. F. & Harrington, R. *Aphids as crop pests*. (CABI, 2007).

[15] Chen Y (2020). An aphid RNA transcript migrates systemically within plants and is a virulence factor. Proc. Natl Acad. Sci. USA.

[16] Margaritopoulos JT, Malarky G, Tsitsipis JA, Blackman RL (2007). Microsatellite DNA and behavioural studies provide evidence of host-mediated speciation in *Myzus persicae* (Hemiptera: Aphididae). Biol. J. Linn. Soc..

[17] Blackman RL (1987). Morphological discrimination of a tobacco-feeding form from *Myzus persicae* (Sulzer) (Hemiptera: Aphididae), and a key to New World *Myzus* (Nectarosiphon) species. Bul. Ent. Res..

[18] von Burg S, Ferrari J, Muller CB, Vorburger C (2008). Genetic variation and covariation of susceptibility to parasitoids in the aphid *Myzus persicae*: no evidence for trade-offs. Proc. R. Soc. Lond. B Biol. Sci..

[19] Bass C (2013). Gene amplification and microsatellite polymorphism underlie a recent insect host shift. Proc. Natl Acad. Sci. USA.

[20] Ramsey JS (2007). Genomic resources for *Myzus persicae*: EST sequencing, SNP identification, and microarray design. BMC Genomics.

[21] Blackman RL (1980). Chromosome numbers in the Aphididae and their taxonomic significance. Syst. Entomol..

[22] Simão FA, Waterhouse RM, Ioannidis P, Kriventseva EV, Zdobnov EM (2015). BUSCO: assessing genome assembly and annotation completeness with single-copy orthologs. Bioinformatics.

[23] Jiang Z (2013). Comparative analysis of genome sequences from four strains of the *Buchnera aphidicola* Mp endosymbion of the green peach aphid, *Myzus persicae*. BMC Genomics.

[24] Guyomar C (2018). Multi-scale characterization of symbiont diversity in the pea aphid complex through metagenomic approaches. Microbiome.

[25] Charlesworth B, Coyne JA, Barton NH (1987). The relative rates of evolution of sex chromosomes and autosomes. Am. Nat..

[26] Oliver KM, Degnan PH, Burke GR, Moran NA (2010). Facultative symbionts in aphids and the horizontal transfer of ecologically important traits. Annu. Rev. Entomol..

[27] Zepeda-Paulo FA (2010). The invasion route for an insect pest species: the tobacco aphid in the New World. Mol. Ecol..

[28] Alexander DH, Lange K (2011). Enhancements to the ADMIXTURE algorithm for individual ancestry estimation. BMC Bioinforma..

[29] Lawson DJ, Hellenthal G, Myers S, Falush D (2012). Inference of population structure using dense haplotype data. PLoS Genet..

[30] Kasprowicz L, Malloch G, Pickup J, Fenton B (2008). Spatial and temporal dynamics of *Myzus persicae* clones in fields and suction traps. Agric. Entomol..

[31] Clements KM (2000). Genetic variation in the *Myzus persicae* complex (Homoptera: Aphididae): evidence for a single species. Ann. Entomol. Soc. Am..

[32] Clements KM, Sorenson CE, Wiegmann BM, Neese PA, Roe RM (2000). Genetic, biochemical, and behavioral uniformity among populations of *Myzus nicotianae* and *Myzus persicae*. Entomol. Exp. Appl..

[33] Garud NR, Messer PW, Buzbas EO, Petrov DA (2015). Recent selective sweeps in North American *Drosophila melanogaster* show signatures of soft sweeps. PLoS Genet..

[34] Gloss AD, Groen SC, Whiteman NK (2016). A genomic perspective on the generation and maintenance of genetic diversity in herbivorous insects. Annu. Rev. Ecol. Evol. Syst..

[35] Simon JC (2015). Genomics of adaptation to host-plants in herbivorous insects. Brief. Funct. Genomics.

[36] Dedryver CA, Le Gallic JF, Mahéo F, Simon JC, Dedryver F (2013). The genetics of obligate parthenogenesis in an aphid species and its consequences for the maintenance of alternative reproductive modes. Heredity.

[37] Margaritopoulos JT, Tsitsipis JA, Goudoudaki S, Blackman RL (2002). Life cycle variation of *Myzus persica*e (Hemiptera: Aphididae) in Greece. Bul. Ent. Res.

[38] Martinez-Torres D, Foster SP, Field LM, Devonshire AL, Williamson MS (1999). A sodium channel point mutation is associated with resistance to DDT and pyrethroid insecticides in the peach-potato aphid, *Myzus persicae* (Sulzer) (Hemiptera: Aphididae). Insect Mol. Biol..

[39] Martinez-Torres D, Devonshire AL, Williamson MS (1997). Molecular studies of knockdown resistance to pyrethroids: cloning of domain II sodium channel gene sequences from insects. Pestic. Sci..

[40] Eleftherianos I, Foster SP, Williamson MS, Denholm I (2008). Characterization of the M918T sodium channel gene mutation associated with strong resistance to pyrethroid insecticides in the peach-potato aphid. Myzus persicae (Sulzer). Bul. Ent. Res..

[41] Fontaine S (2011). Uncommon associations in target resistance among French populations of *Myzus persicae* from oilseed rape crops. Pest Manag. Sci..

[42] Andrews MC, Callaghan A, Field LM, Williamson MS, Moores GD (2004). Identification of mutations conferring insecticide-insensitive AChE in the cotton-melon aphid, *Aphis gossypii* Glover. Insect Mol. Biol..

[43] Nabeshima T, Kozaki T, Tomita T, Kono Y (2003). An amino acid substitution on the second acetylcholinesterase in the pirimicarb-resistant strains of the peach potato aphid, *Myzus persicae*. Biochem. Biophys. Res. Commun..

[44] Anthony N, Unruh T, Ganser D, ffrench-Constant R (1998). Duplication of the *Rdl* GABA receptor subunit gene in an insecticide-resistant aphid, *Myzus persicae*. Mol. Gen. Genet..

[45] Bass C (2011). Mutation of a nicotinic acetylcholine receptor β subunit is associated with resistance to neonicotinoid insecticides in the aphid *Myzus persicae*. BMC Neurosci..

[46] Singh KS (2020). The genetic architecture of a host shift: an adaptive walk protected an aphid and its endosymbiont from plant chemical defences. Sci. Adv..

[47] Panini M, Dradi D, Marani G, Butturini A, Mazzoni E (2014). Detecting the presence of target-site resistance to neonicotinoids and pyrethroids in Italian populations of *Myzus persicae*. Pest Manag. Sci..

[48] Roy L, Fontaine S, Caddoux L, Micoud A, Simon JC (2013). Dramatic changes in the genotypic frequencies of target insecticide resistance in French populations of *Myzus persicae* (Hemiptera: Aphididae) over the last decade. J. Econ. Entomol..

[49] Sonoda S (2012). Frequencies of the M918I mutation in the sodium channel of the diamondback moth in China, Thailand and Japan and its association with pyrethroid resistance. Pest. Biochem. Physiol..

[50] Dang K (2015). Identification of putative kdr mutations in the tropical bed bug, *Cimex hemipterus* (Hemiptera: Cimicidae). Pest Manag. Sci..

[51] Vais H, Williamson MS, Devonshire AL, Usherwood PNR (2001). The molecular interactions of pyrethroid insecticides with insect and mammalian sodium channels. Pest Manag. Sci..

[52] de Little SC, Umina PA (2017). Susceptibility of Australian *Myzus persicae* (Hemiptera: Aphididae) to three recently registered insecticides: Spirotetramat, cyantraniliprole, and sulfoxaflor. J. Econ. Entomol..

[53] Voudouris CC (2017). Evolution of imidacloprid resistance in *Myzus persicae* in Greece and susceptibility data for spirotetramat. Pest Manag. Sci..

[54] Lueke B (2020). Identification and functional characterization of a novel acetyl-CoA carboxylase mutation associated with ketoenol resistance in *Bemisia tabaci*. Pest. Biochem. Physiol..

[55] Guest M, Kriek N, Flemming AJ (2020). Studies of an insecticidal 1 inhibitor of acetyl-CoA carboxylase in the nematode *C. elegans*. Pest. Biochem. Physiol..

[56] ffrench-Constant R (2013). The molecular genetics of insecticide resistance. Genetics.

[57] Roush RT, McKenzie JA (1987). Ecological genetics of insecticide and acaricide resistance. Annu. Rev. Entomol..

[58] Robin C, Battlay P, Fournier-Level A (2018). What can genetic association panels tell us about evolutionary processes in insects?. Curr. Opin. Insect Sci..

[59] Weetman D (2010). Association mapping of insecticide resistance in wild *Anopheles gambiae* populations: major variants identified in a low-linkage disequilbrium genome. PLoS ONE.

[60] Whitfield CW (2006). Thrice out of Africa: ancient and recent expansions of the honey bee, *Apis mellifera*. Science.

[61] Mackay TF (2012). The *Drosophila melanogaster* genetic reference panel. Nature.

[62] Margaritopoulos JT, Blackman RL, Tsitsipis JA, Sannino L (2003). Co-existence of different host-adapted forms of the *Myzus persicae* group (Hemiptera: Aphididae) in southern Italy. Bul. Ent. Res.

[63] Drès M, Mallet J (2002). Host races in plant-feeding insects and their importance in sympatric speciation. Philos. Trans. R. Soc. B Biol. Sci..

[64] Krueger, F. A wrapper tool around Cutadapt and FastQC to consistently apply quality and adapter trimming to FastQ files. http://www.bioinformatics.babraham.ac.uk/projects/trim_galore/ (2015).

[65] Koren S (2017). Canu: scalable and accurate long-read assembly via adaptive k-mer weighting and repeat separation. Genome Res.

[66] Ruan J, Li H (2020). Fast and accurate long-read assembly with wtdbg2. Nat. Methods.

[67] Kolmogorov M, Yuan J, Lin Y, Pevzner PA (2019). Assembly of long, error-prone reads using repeat graphs. Nat. Biotechnol..

[68] Chin C-S (2016). Phased diploid genome assembly with single-molecule real-time sequencing. Nat. Methods.

[69] Chakraborty M, Baldwin-Brown JG, Long AD, Emerson JJ (2016). Contiguous and accurate de novo assembly of metazoan genomes with modest long read coverage. Nucleic Acids Res..

[70] Mapleson D, Accinelli GG, Kettleborough G, Wright J, Clavijo BJ (2017). KAT: a K-mer analysis toolkit to quality control NGS datasets and genome assemblies. Bioinformatics.

[71] Vaser R, Sović I, Nagarajan N, Šikić M (2017). Fast and accurate de novo genome assembly from long uncorrected reads. Genome Res.

[72] Walker BJ (2014). Pilon: An integrated tool for comprehensive microbial variant detection and genome assembly improvement. PLoS ONE.

[73] Roach MJ, Schmidt SA, Borneman AR (2018). Purge Haplotigs: allelic contig reassignment for third-gen diploid genome assemblies. BMC Bioinforma..

[74] Lieberman-Aiden E (2009). Comprehensive mapping of long-range interactions reveals folding principles of the human genome. Science.

[75] Durand NC (2016). Juicer provides a one-click system for analyzing loop-resolution Hi-C experiments. Cell Syst..

[76] Dudchenko O (2017). De novo assembly of the *Aedes aegypti* genome using Hi-C yields chromosome-length scaffolds. Science.

[77] Dudchenko, O. *et al*. The Juicebox Assembly Tools module facilitates de novo assembly of mammalian genomes with chromosome-length scaffolds for under $1000. *bioRxiv*10.1101/254797 (2018).

[78] Laetsch DR, Blaxter ML (2017). BlobTools: Interrogation of genome assemblies. F1000Res..

[79] Shen W, Le S, Li Y, Hu F (2016). SeqKit: A cross-platform and ultrafast toolkit for FASTA/Q file manipulation. PLoS ONE.

[80] Smit, A. F. A. & Hubley, R. RepeatModeler Open-1.0. http://www.repeatmasker.org (2010).

[81] Borodovsky, M. & Lomsadze, A. Eukaryotic gene prediction using GeneMark.hmm-E and GeneMark-ES. *Curr. Protoc. Bioinformatics* Chapter 4, Unit-4.6.10, 10.1002/0471250953.bi0406s35 (2011). 10.1002/0471250953.bi0406s35 PMC3204378 21901742

[82] Stanke M, Morgenstern B (2005). AUGUSTUS: a web server for gene prediction in eukaryotes that allows user-defined constraints. Nucleic Acids Res..

[83] Hoff KJ, Lange S, Lomsadze A, Borodovsky M, Stanke M (2015). BRAKER1: unsupervised RNA-Seq-based genome annotation with GeneMark-ET and AUGUSTUS. Bioinformatics.

[84] Kim D, Paggi JM, Park C, Bennett C, Salzberg SL (2019). Graph-based genome alignment and genotyping with HISAT2 and HISAT-genotype. Nat. Biotechnol..

[85] Li H (2009). The Sequence Alignment/Map format and SAMtools. Bioinformatics.

[86] Panini M (2021). Transposon-mediated insertional mutagenesis unmasks recessive insecticide resistance in the aphid *Myzus persicae*. Proc. Natl Acad. Sci. USA.

[87] Andrews, S. FastQC: a quality control tool for high throughput sequence data. *Available online at**:* http://www.bioinformatics.babraham.ac.uk/projects/fastqc (2010).

[88] Voronova, N. V. et al. Characteristic and variability of five complete aphid mitochondrial genomes: Aphis fabae mordvilkoi, Aphis craccivora, Myzus persicae, Therioaphis tenera and Appendiseta robiniae (Hemiptera; Sternorrhyncha; Aphididae. Int. J. Biol. Macromol. 149, 187–206 (2020). . 10.1016/j.ijbiomac.2019.12.276 31917211

[89] Li, H. Aligning sequence reads, clone sequences and assembly contigs with BWA-MEM. *arXiv***1303.3997v1 [q-bio.GN]**. (2013).

[90] Van der Auwera GA (2013). From FastQ data to high confidence variant calls: the Genome Analysis Toolkit best practices pipeline. Curr. Protoc. Bioinforma..

[91] Bolger AM, Lohse M, Usadel B (2014). Trimmomatic: A flexible trimmer for Illumina sequence data. Bioinformatics.

[92] Wood DE, Lu J, Langmead B (2019). Improved metagenomic analysis with Kraken 2. Genome Biol..

[93] Kim D, Song L, Breitwieser FP, Salzberg SL (2016). Centrifuge: rapid and sensitive classification of metagenomic sequences. Genome Res..

[94] Nguyen LT, Schmidt HA, Haeseler AV, Minh BQ (2015). IQ-TREE: A fast and effective stochastic algorithm for estimating maximum likelihood phylogenies. Mol. Biol. Evol..

[95] Huson DH, Bryant D (2006). Application of phylogenetic networks in evolutionary studies. Mol. Biol. Evol..

[96] Purcell S (2007). PLINK: a toolset for whole-genome association and population-based linkage analysis. Am. J. Hum. Genet..

[97] Dray S, Dufour AB (2007). The ade4 package: implementing the duality diagram for ecologists. J. Stat. Softw..

[98] Excoffier L, Smouse PE, Quattro J (1992). Analysis of molecular variance inferred from metric distances among DNA haplotypes: application to human mitochondrial DNA restriction data. Genetics.

[99] Excoffier L, Lischer H (2010). Arlequin suite ver 3.5: A new series of programs to perform population genetics analyses under Linux and Windows. Mol. Ecol. Resour..

[100] Danecek P (2011). 1000 Genomes Project Analysis Group, The variant call format and VCFtools. Bioinformatics.

[101] Howey, R. & Cordell, H. J. MapThin https://www.staff.ncl.ac.uk/richard.howey/mapthin/introduction.html (2011).

[102] Barrett JC, Fry B, Maller J, Daly MJW (2005). Haploview: analysis and visualization of LD and haplotype maps. Bioinformatics.

[103] Han E, Sinsheimer JS, Novembre J (2015). Fast and accurate site frequency spectrum estimation from low coverage sequence data. Bioinformatics.

[104] Li H (2011). A statistical framework for SNP calling, mutation discovery, association mapping and population genetical parameter estimation from sequencing data. Bioinformatics.

[105] Katoh K, Misawa K, Kuma K-I, Miyata T (2002). MAFFT: a novel method for rapid multiple sequence alignment based on fast Fourier transform. Nucleic Acids Res..

[106] Clement M, Posada D, Crandall KA (2000). TCS: a computer program to estimate gene genealogies. Mol. Ecol..

[107] Singh, K. S., & Bass, C. Bioproject PRJNA 574571. National Center for Biotechnology Information. https://www.ncbi.nlm.nih.gov/bioproject/PRJNA574571. Deposited 30 December 2020.

[108] Singh, K. S., & Bass, C. Genome assembly: Myzus persicae G006 genome v3.0. AphidBase. https://bipaa.genouest.org/is/aphidbase/. Deposited 30 March 2020.

