# Supplementary material for: Global patterns in genomic diversity underpinning the evolution of insecticide resistance in the aphid crop pest Myzuspersicae
Source: Commun Biol. 2021 Jul 7;4:847. doi: 10.1038/s42003-021-02373-x (PMC8263593; doi:10.1038/s42003-021-02373-x)
Supplement: Supplementary file 1 — Supplemental Material [file 42003_2021_2373_MOESM1_ESM.pdf]

Supplementary Table 1 Assembly properties of the existing (G006v1) and new (G006v2) *Myzus persicae* genomes.

Assembly	G006v1	G006v2
Sequencing approach	Illumina PE + MP	PacBio + Hi-C
Number of total contigs	6105	773
Number of total Scaffolds	4022	336
Contig N50 (bp)	213873	3162279
Contig L50	475	32
Longest Contig (bp)	1546484	18208374
Scaffold N50 (bp)	435781	69950500
Scaffold L50	223	2
Longest Scaffold (bp)	2199663	99459606
Total assembled size (bp)	347304760	390790610
GC content	30.02%	30.02%
Total Complete BUSCOs	98.4%	97.3%
Total Duplicated BUSCOs	2.9%	2.5%
Total Missing BUSCOs	0.9%	2.2%
Total gene content	18529	23214
Total gene annotated	18433	21899

Supplementary Table 2 Hierarchical analysis of molecular variance (AMOVA) of *M. persicae* grouped by A) host and B) geographic region. Host groups comprised peach, tobacco, pepper, oilseed rape, and geographic region Europe, Africa, Asia, Australia, Asia, South America, and North America.

Source of variation	Sum of squares	Variance components	% variation	Fixation indices	*P* value
**A**					
Among Groups	60491.804	331.658	6.31	F_{CT} = 0.063	< 0.001
Among Populations Within Groups	143072.599	370.49	7.05	F_{SC} = 0.075	< 0.001
Within populations	710443.324	4554.124	86.64	F_{ST} = 0.134	< 0.001
**B**					
Among Groups	72021.453	299.128	5.63	F_{CT} = 0.056	< 0.001
Among Populations Within Groups	258650.099	369.804	6.95	F_{SC} = 0.074	< 0.001
Within populations	892457.699	4648.217	87.42	F_{ST} = 0.126	< 0.001

Supplementary Table 3 Summary of linkage disequilibrium (LD) blocks across the five autosomes of *M. persicae* clones from peach and tobacco. Due to sample size LD blocks could not be estimated independently for populations from tobacco in Greece and Italy.

Host	Peach										Tobacco				
**Country**	**Greece**					**Italy**					**Greece + Italy**				
**Autosome**	2	3	4	5	6	2	3	4	5	6	2	3	4	5	6
**#LD blocks**	750	541	472	242	245	409	162	260	93	117	2306	730	1396	683	716
**Avg. Len. (Kb)**	13.9	12.5	12	14.8	14.7	10.15	14.7	11.5	10.1	11.7	6.9	16.3	6.7	7.5	6.8
**Shortest (Kb)**	0.935	0.337	0.629	1.058	1.09	0.481	0.977	1.091	1.181	1.231	0.002	0.757	0.002	0.002	0.002
**Longest (Kb)**	536.8	184	156.8	169	168.4	134.1	193.128	78.5	65	80.614	133.2	426.4	128.1	147.8	143.5

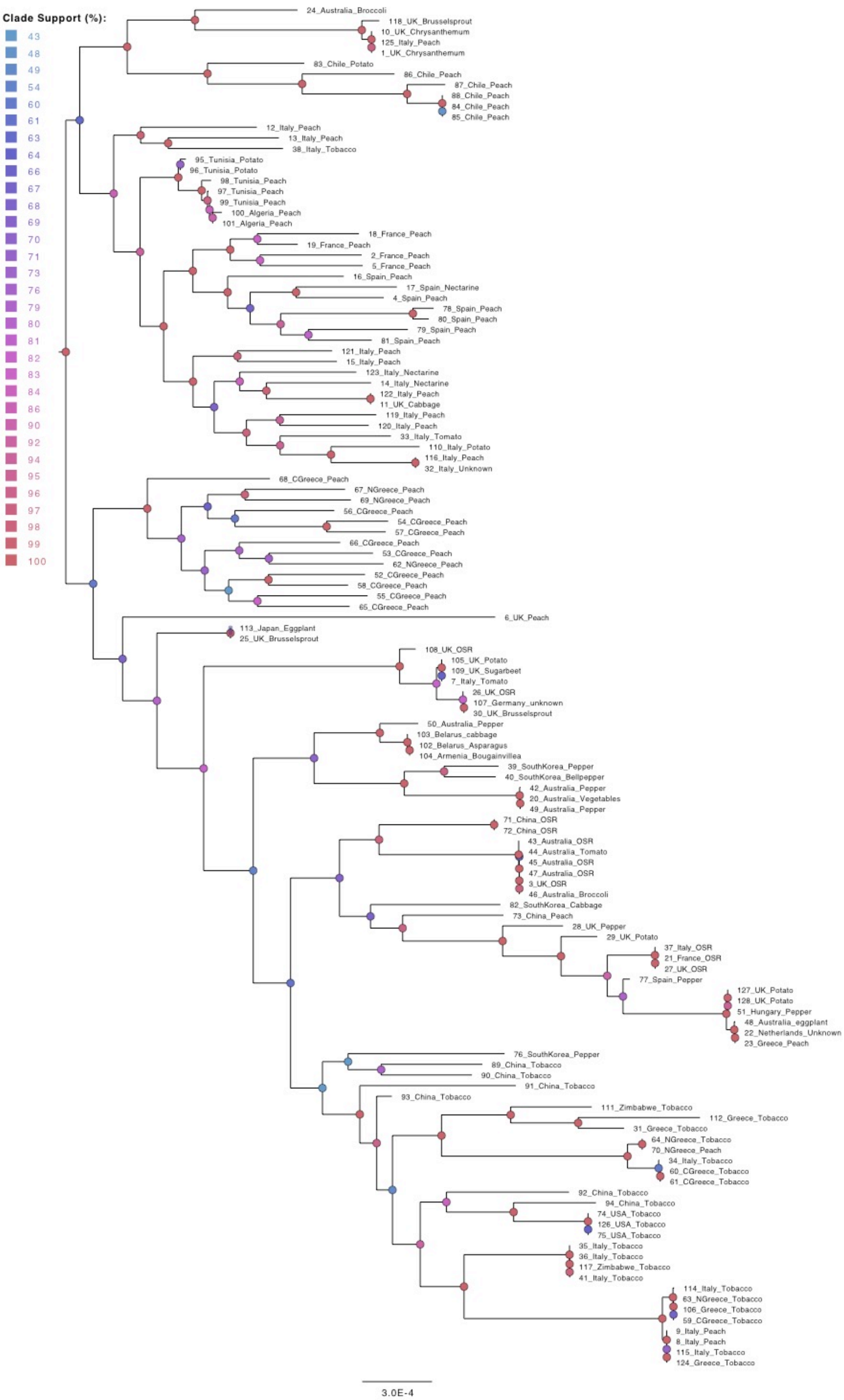

Supplementary Fig. 1 Phylogram depicting a maximum likelihood phylogeny of 127 *M. persicae* clones based on >1M biallelic SNPs. *Myzus cerasi* was used as an outgroup (not shown in the tree).

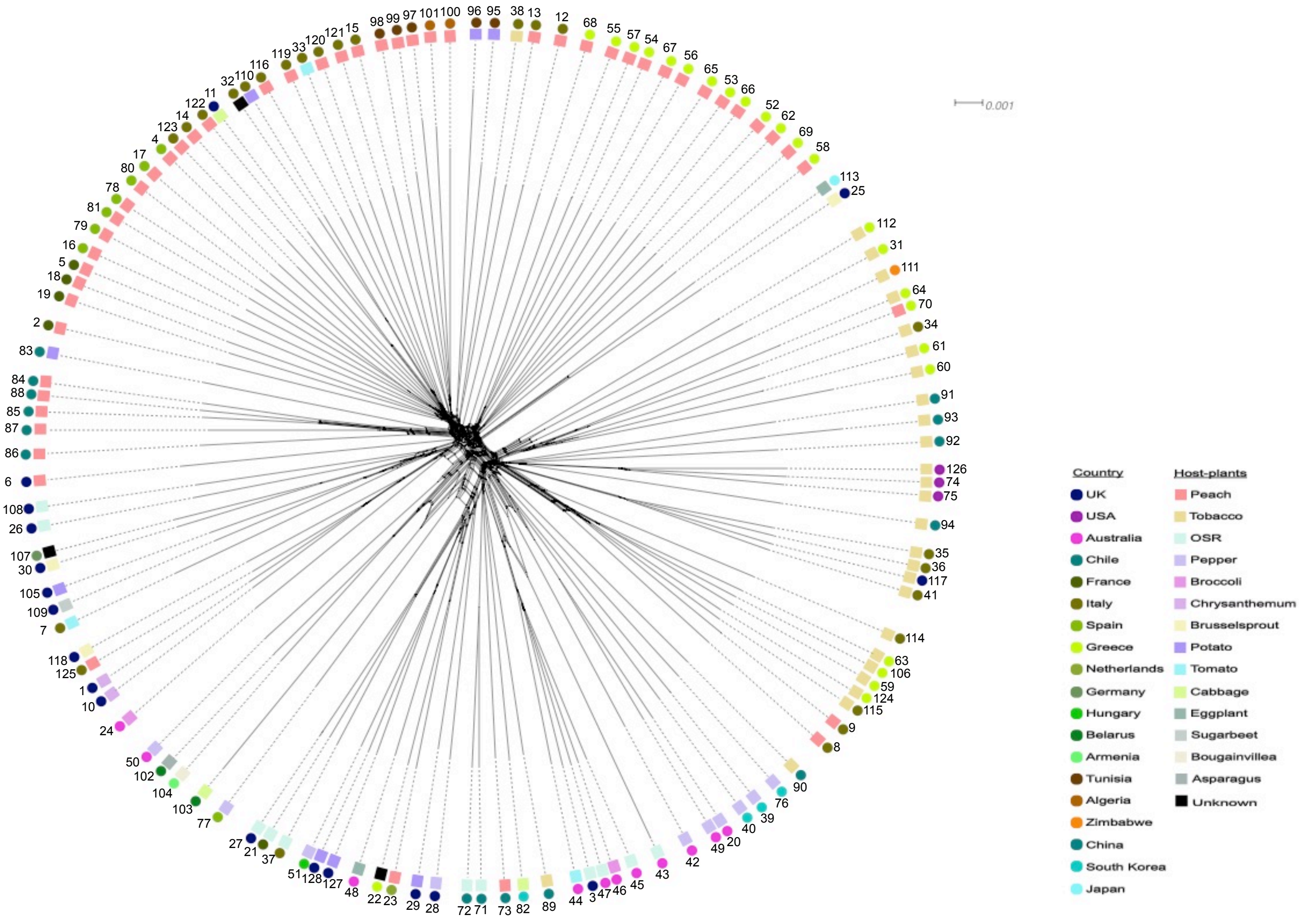

Supplementary Fig. 2 Phylogenetic split network of 127 *M. persicae* clones based on >1M biallelic SNPs generated using the neighbor-net algorithm. The geographic origin of clones and the host plant from which they were collected are indicated by coloured circles and squares respectively. *Myzus cerasi* was used as an outgroup (not shown). Clone identification numbers (corresponding to Supplementary Data 1) are also included.

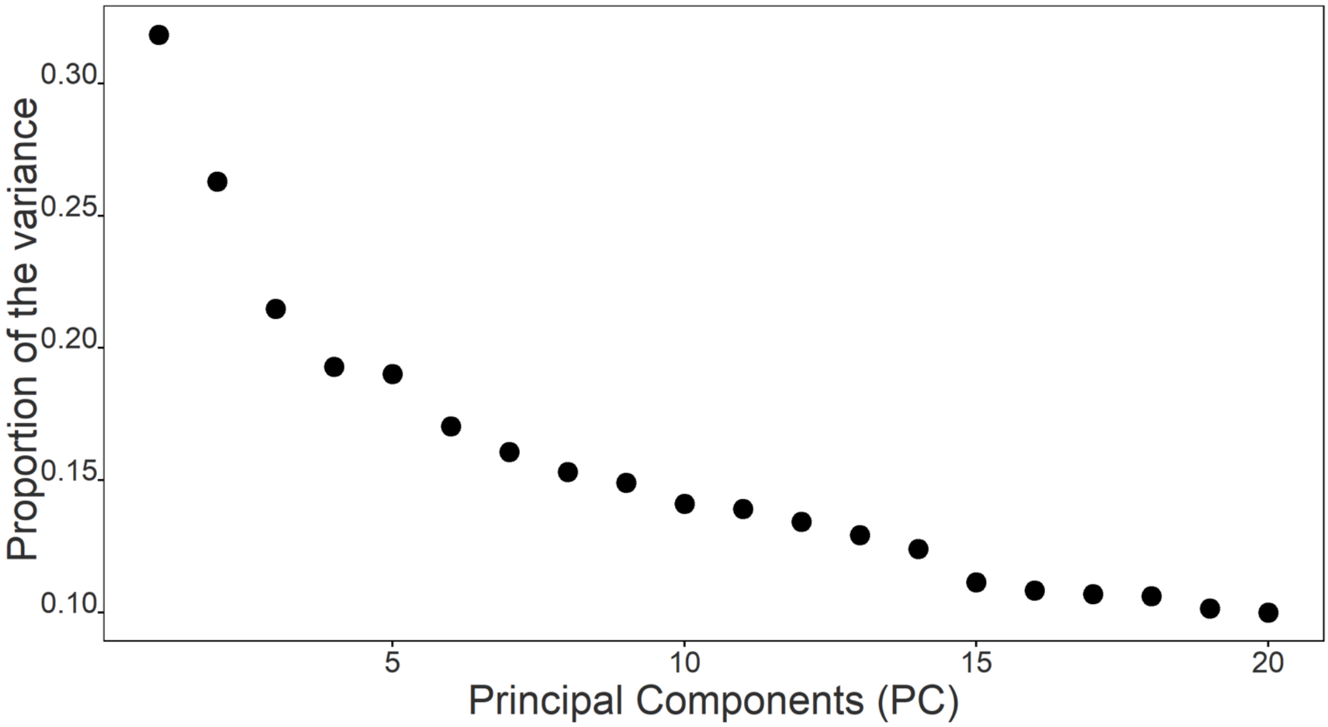

Supplementary Fig. 3 Plot of the proportion of variance explained by the first 20 principal components in PCA of genetic diversity of 127 sequenced clones of *M. persicae*.

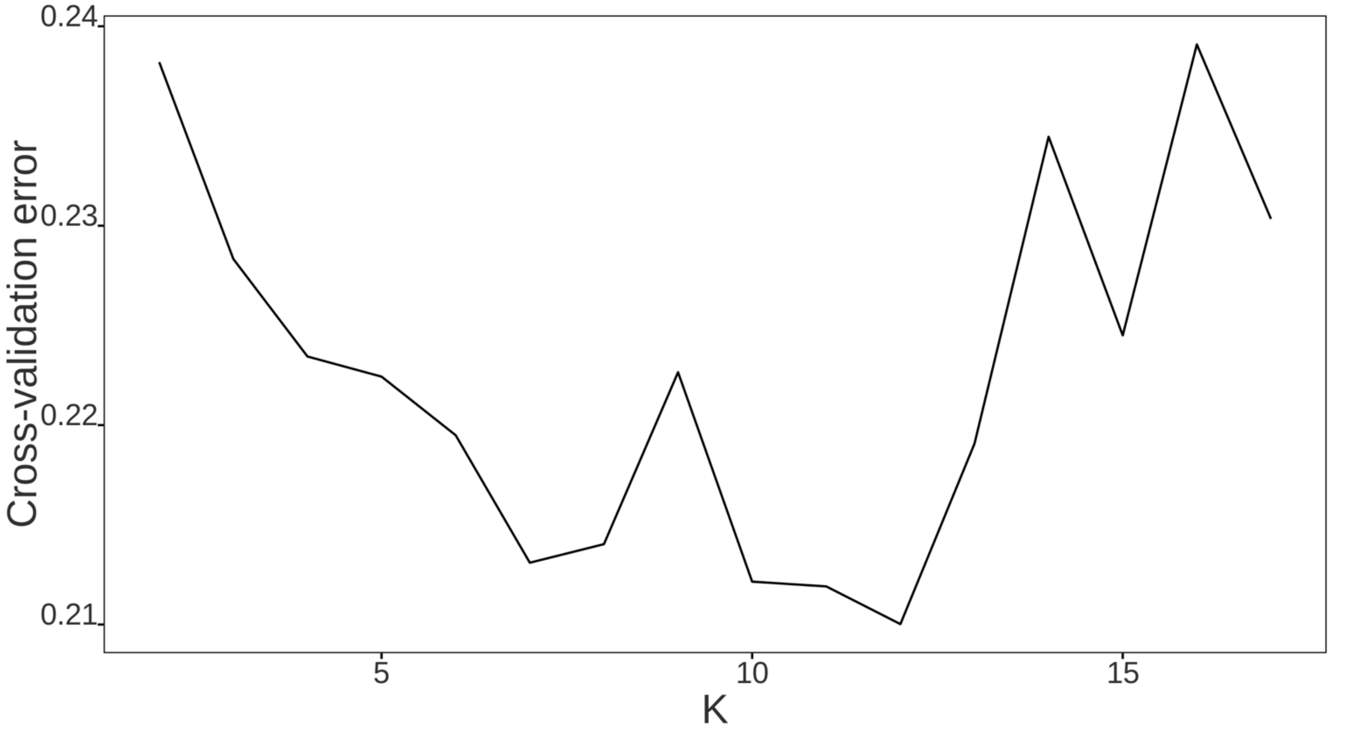

Supplementary Fig. 4 Plot of ADMIXTURE cross validation error from K=2 through K=20.

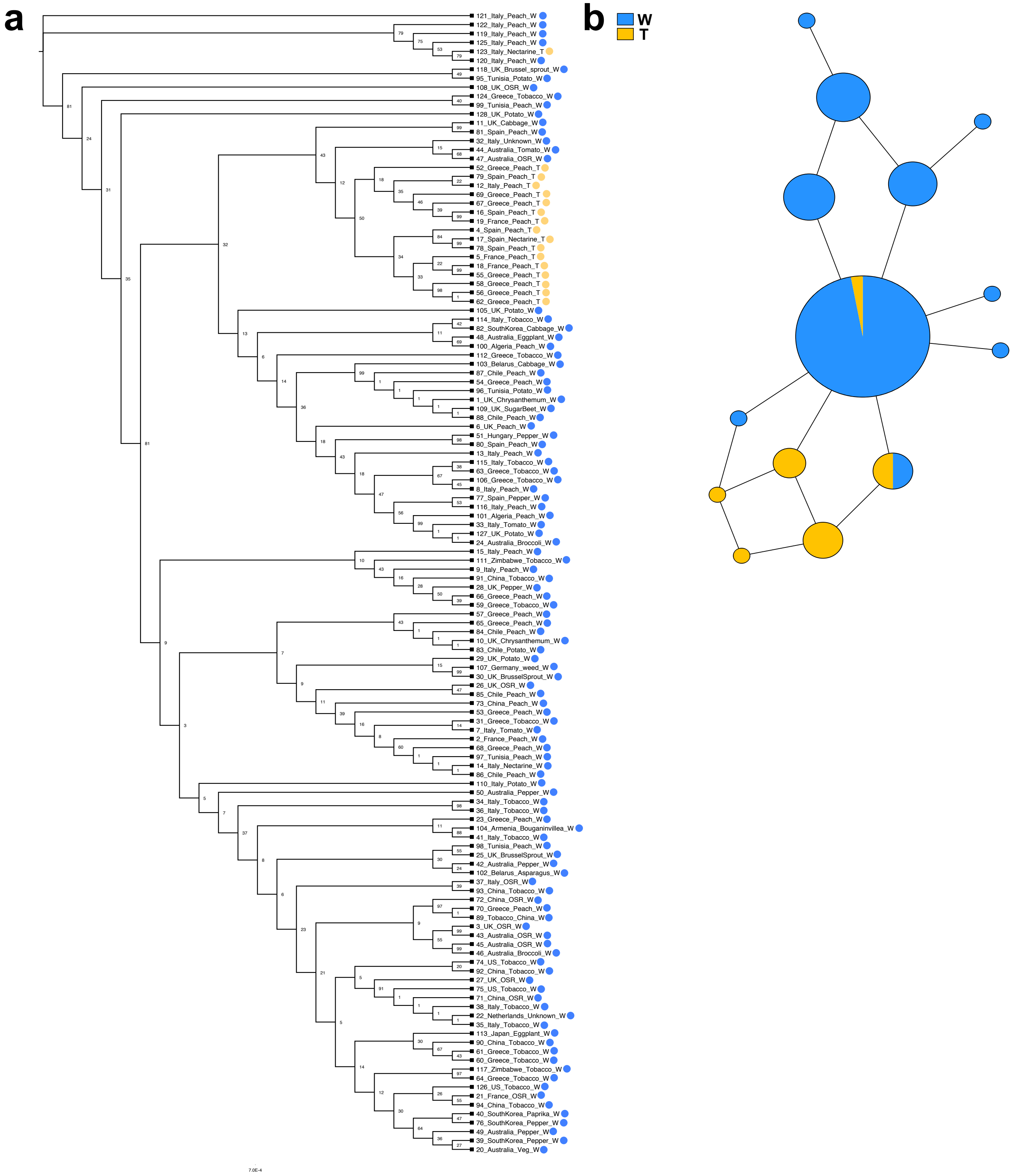

Supplementary Fig. 5 Haplotype analysis of the nicotinic acetylcholine receptor (nAChR) β1 subunit. a Maximum likelihood phylogenetic tree of nAChR β1 haplotypes in the sampled *M. persicae* clones. The mutation profile of each haplotype is indicated using coloured circles. **b** Haplotype network calculated by the TCS method.

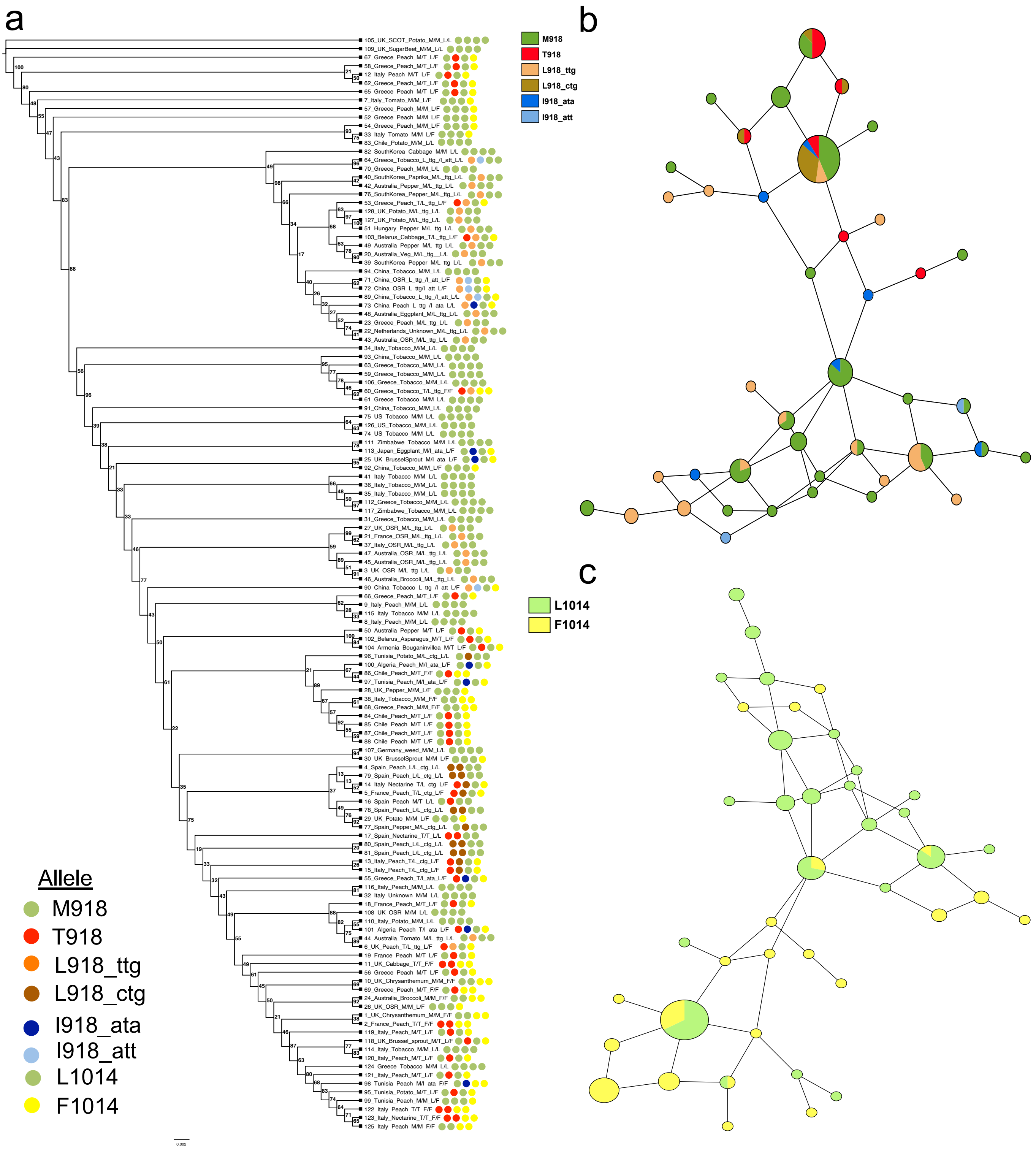

Supplementary Fig. 6 Haplotype analysis of the voltage-gated sodium channel (VGSC). a Maximum likelihood phylogenetic tree of VGSC haplotypes in the sampled *M. persicae* clones. The mutation profile of each haplotype is indicated using coloured circles, with the first two coloured circles representing the two alleles at amino acid position 918 (skdr position) and the third and fourth coloured circles representing the two alleles at amino acid position 1014 (kdr position). **b, c** Haplotype network calculated by the TCS method for skdr (b) and kdr (c) mutations.

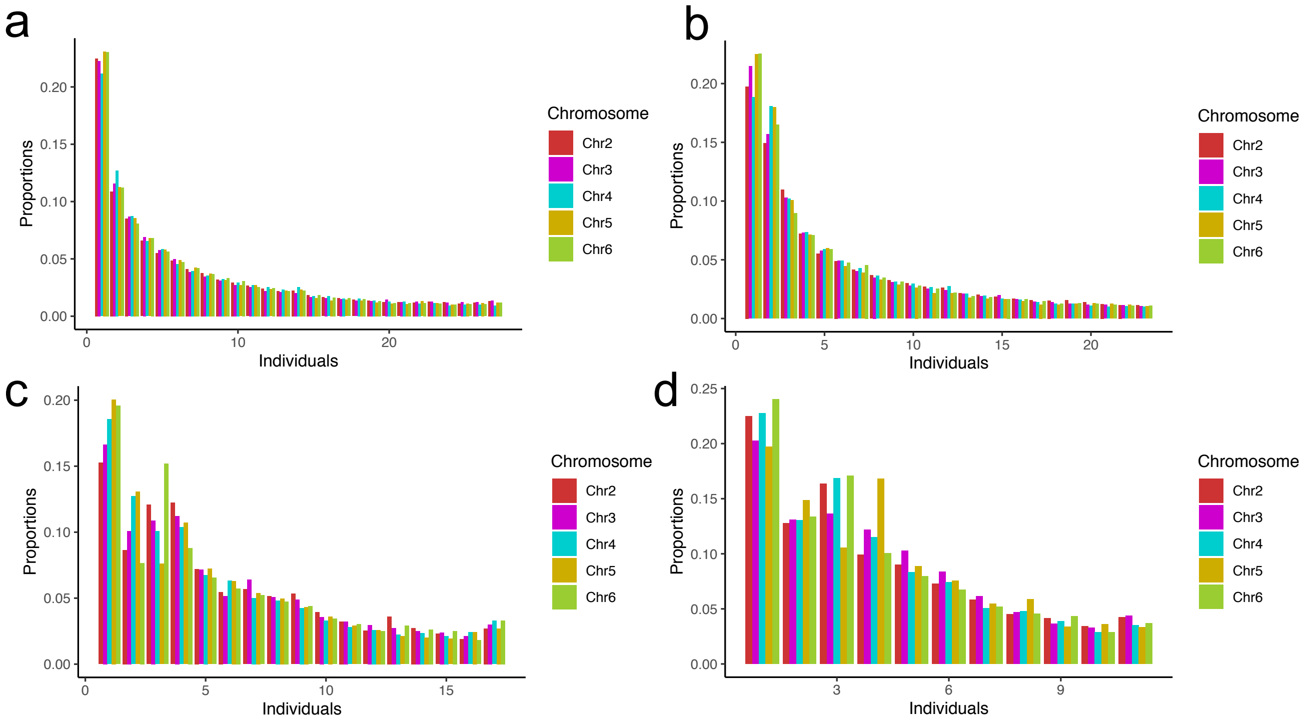

Supplementary Fig. 7 Site frequency spectrum (SFS) analysis of *M. persicae* from peach and tobacco in Italy and Greece. The SFS for all autosomes of clones from **a** peach-Greece, **b** peach-Italy, **c** tobacco-Greece and **d** tobacco-Italy is shown.

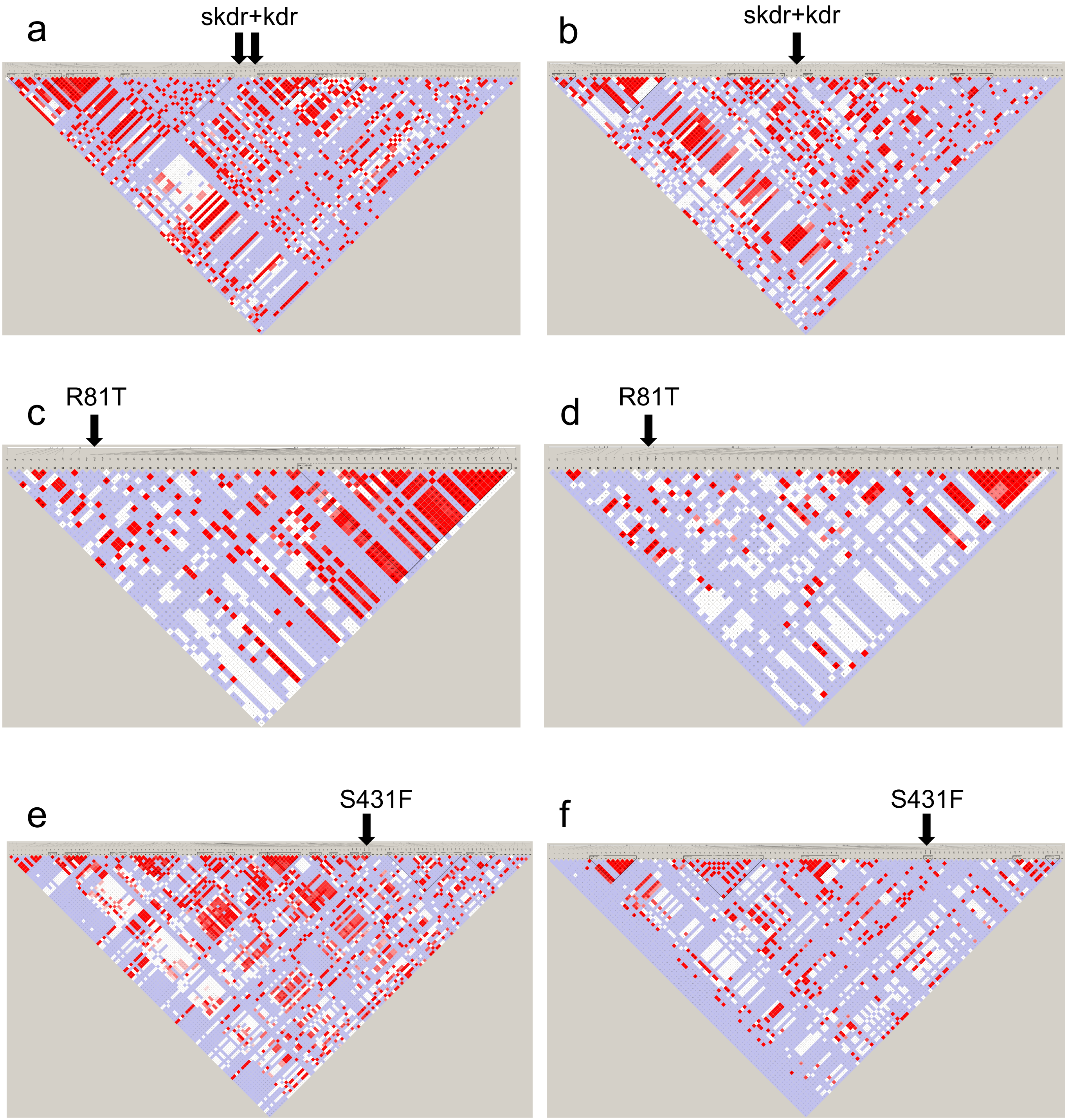

Supplementary Fig. 8 Short-range linkage disequilibrium (LD) at three insecticide resistance loci in *M. persicae* from peach and tobacco. Grid plots are shown for the loci encompassing the sites of the kdr+skdr mutations in *M. persicae* from **a** peach and **b** tobacco, the R81T mutation in clones from **c** peach and **d** tobacco, and the S431F mutation in clones from **e** peach and **f** tobacco. Each square corresponds to one LD value (r^2) between 2 variant sites using a colour code from a complete lack of LD (r^2 = 0: white) to perfect LD (r^2 = 1: red). The positions of insecticide resistance-associated mutations are highlighted above each grid plot using black arrows.
