# Supplementary material for: Global patterns in genomic diversity underpinning the evolution of insecticide resistance in the aphid crop pest Myzuspersicae
Source: Commun Biol. 2021 Jul 7;4:847. doi: 10.1038/s42003-021-02373-x (PMC8263593; doi:10.1038/s42003-021-02373-x)
Supplement: Supplementary file 2 — Description of Supplementary Files [file 42003_2021_2373_MOESM2_ESM.pdf]

Description of Additional Supplementary Files

File name: Supplementary Data 1

Description: *Details of the Myzus persicae clones included in this study.* Table includes information on the geographic origin and host plant from which each clone was collected (where known), SRA accession numbers and sequence coverage, and genotype of each clone for known resistance mutations. The latter include the nicotinic acetylcholine receptor mutation R81T that's confers high-level resistance to neonicotinoids; the voltage-gated sodium channel (VGSC) knock-down resistance (*kdr*) mutations L1014F, M918T, M918L, and M918I that lead to pyrethroid resistance; the acetylcholinesterase enzyme mutation S431F, conferring resistance to dimethylcarbamates; the γ-aminobutyric acid (GABA) receptor mutation G302, conferring resistance to cylodiene insecticides; and the T74I mutation that is diagnostic for amplification of the P450 gene *CYP6CY3*, which confers moderate levels of resistance to neonicotinoids. The presence of the *CYP6CY3* amplification breakpoint is also indicated. In all cases only deviations from wild-type are indicated (i.e. blank cells indicate wild-type), and the star symbol is used to indicate clones that are homozygous for a given mutation. For M918I and M918L, the two alternative codons resulting in these substitutions observed in each clone is indicated.

File name: Supplementary Data 2

Description: *Pairwise divergence matrix of sequenced M. persicae clones*. The matrix was estimated using the *dist()* function of the R software environment -v 3.6.1**.**

File name: Supplementary Data 3

Description: *Occurrence of bacterial endosymbionts and other microbes in the sequenced M. persicae clones*. The first tab displays the results of mapping sequence data to a collection of >30 reference genomes of known aphid symbionts, their associated plasmids, and a number of viruses known to infect aphids. Unmapped reads from this analyses were subsequently submitted to the Kraken2 and Centifuge databases to identify other microbial sequences, with results displayed in the second and third tabs respectively.

File name: Supplementary Data 4

Description: Genes located within putative selection sweeps identified by H12 analysis.
