# Supplementary material for: Global patterns in genomic diversity underpinning the evolution of insecticide resistance in the aphid crop pest Myzuspersicae
Source: Commun Biol. 2021 Jul 7;4:847. doi: 10.1038/s42003-021-02373-x (PMC8263593; doi:10.1038/s42003-021-02373-x)
Supplement: Supplementary file 7 — Reporting Summary [file 42003_2021_2373_MOESM7_ESM.pdf]

nature research

Corresponding author(s): Chris Bass

Last updated by author(s): Jun 6, 2021

Reporting Summary

Nature Research wishes to improve the reproducibility of the work that we publish. This form provides structure for consistency and transparency in reporting. For further information on Nature Research policies, see our Editorial Policies and the Editorial Policy Checklist.

Statistics

For all statistical analyses, confirm that the following items are present in the figure legend, table legend, main text, or Methods section.

n/a	Confirmed	
☐	☒	The exact sample size (*n*) for each experimental group/condition, given as a discrete number and unit of measurement
☐	☒	A statement on whether measurements were taken from distinct samples or whether the same sample was measured repeatedly
☐	☒	The statistical test(s) used AND whether they are one- or two-sided<br>*Only common tests should be described solely by name; describe more complex techniques in the Methods section.*
☒	☐	A description of all covariates tested
☒	☐	A description of any assumptions or corrections, such as tests of normality and adjustment for multiple comparisons
☐	☒	A full description of the statistical parameters including central tendency (e.g. means) or other basic estimates (e.g. regression coefficient) AND variation (e.g. standard deviation) or associated estimates of uncertainty (e.g. confidence intervals)
☒	☐	For null hypothesis testing, the test statistic (e.g. *F*, *t*, *r*) with confidence intervals, effect sizes, degrees of freedom and *P* value noted<br>*Give P values as exact values whenever suitable.*
☒	☐	For Bayesian analysis, information on the choice of priors and Markov chain Monte Carlo settings
☒	☐	For hierarchical and complex designs, identification of the appropriate level for tests and full reporting of outcomes
☒	☐	Estimates of effect sizes (e.g. Cohen's *d*, Pearson's *r*), indicating how they were calculated

Our web collection on statistics for biologists contains articles on many of the points above.

Software and code

Policy information about availability of computer code

Data collection	Microsoft Excel (2011 Version 14.7.2)
Data analysis	Biomatters Ltd Geneious Version 10.2.3; Microsoft Excel 2011 -v14.7.2; TrimGalore -v 0.4.0; Canu -v1.8.0; wtdbg2 -v1.0; Flye -v2.6.0; quickmerge -v0.3; KAT -v1.0.0; BUSCO -v4.14; Racon -v1.3.1; Pilon -v1.22; Purge_haplotigs -v1.0.4; Juicer -v1.6; 3D de novo assembly (3D-DNA) pipeline -v180419; BlobTools -v1.1, SeqKit v0.9.1; RepeatMasker -v4.0.7; RepeatModeler -v2.0.2; GeneMark-ES –v4.3.8, AUGUSTUS –v3.3.0; BRAKER -v2.1.2; HISAT2 v2.0.5; SAMtools -v1.3 and -v1.90; BLAST2GO –v5.2.5; BWA -v 0.7.17; GATK -v 4.1.0; Trimmomatic -v0.33; Kraken2 -v2.1.1; Centrifuge -v1.0.4; IQTree -v 1.6; SplitsTree -v4.16.1; Plink -v1.9; ade4 -v1.7-16; ADMIXTURE -v1.3.0; fineSTRUCTURE -v2; Arlequin v. 3.52; VCFtools -v0.1.14; SelectionHapStats -v1; bcftools -v 1.9; MAFFT -v7.471; TCS -v1.21; MapThin -v1.11; ANGSD -v0.921; HaploView -v02032021; R

For manuscripts utilizing custom algorithms or software that are central to the research but not yet described in published literature, software must be made available to editors and reviewers. We strongly encourage code deposition in a community repository (e.g. GitHub). See the Nature Research guidelines for submitting code & software for further information.

Data

Policy information about availability of data

All manuscripts must include a data availability statement. This statement should provide the following information, where applicable:

- Accession codes, unique identifiers, or web links for publicly available datasets
- A list of figures that have associated raw data
- A description of any restrictions on data availability

The sequence data generated in this study has been deposited with NCBI under the Bio Project ID: PRJNA574571. For individual accession numbers associated with each clone see Supplementary Data 1. The genome assembly of M. persicae clone G006 is also available at AphidBase https://bipaa.genouest.org/is/aphidbase/.

Field-specific reporting

Please select the one below that is the best fit for your research. If you are not sure, read the appropriate sections before making your selection.

☐ Life sciences ☐ Behavioural & social sciences ☒ Ecological, evolutionary & environmental sciences

For a reference copy of the document with all sections, see nature.com/documents/nr-reporting-summary-flat.pdf

Ecological, evolutionary & environmental sciences study design

All studies must disclose on these points even when the disclosure is negative.

Study description	The aphid Myzus persicae is a destructive agricultural pest that displays an exceptional ability to develop resistance to both natural and synthetic insecticides. In this study we generated a population genomic resource for M. persicae comprising a high-quality chromosomal-scale genome assembly together with resequenced genomes of 127 clonal aphid lines collected from all continents where crops are grown. We used this data to investigate the mechanisms underpinning insecticide resistance, and ecological and evolutionary factors influencing its emergence and spread.
Research sample	The research sample comprised 127 clones (female asexual lineages) of Myzus persicae s.l. derived from 19 countries covering all continents except Antarctica (see Supplementary Table 2 of the manuscript for full details on the clone library). The clones were collected from 14 host plants encompassing a range of agriculturally important crops. Of the assembled clones, ~110 are maintained as asexual lineages in the Bass laboratory. These are available to other researchers as live cultures or preserved material. All clones were sequenced using Illumina paired-end sequencing to an average coverage of 40X (see Supplementary Table 2).
Sampling strategy	Samples were collected from the laboratories of the co-authours and was thus opportunistic. However, our aim was to sample as geographically widely as possible and from a range of agriculturally important host plants. Any sampling limitations in regards to specific host plants are fully acknowledged in the manuscript during discussion of the results.
Data collection	All clones were sequenced using Illumina paired-end sequencing to an average coverage of 40X (see Supplementary Table 2) and data was collected and held by the laboratory of the corresponding author and distributed to co-authors as required. All other data was collected in the laboratory of the corresponding author.
Timing and spatial scale	Data was collected from 2015-2020 by the laboratory of the corresponding author.
Data exclusions	No data was excluded from any analyses
Reproducibility	All attempts to repeat experiments described in this study were successful.
Randomization	Not applicable to this study
Blinding	Not applicable to this study

Did the study involve field work? ☐ Yes ☒ No

Reporting for specific materials, systems and methods

We require information from authors about some types of materials, experimental systems and methods used in many studies. Here, indicate whether each material, system or method listed is relevant to your study. If you are not sure if a list item applies to your research, read the appropriate section before selecting a response.

Materials & experimental systems

n/a	Involved in the study	
☒	☐	Antibodies
☒	☐	Eukaryotic cell lines
☒	☐	Palaeontology and archaeology
☐	☒	Animals and other organisms
☒	☐	Human research participants
☒	☐	Clinical data
☒	☐	Dual use research of concern

Methods

n/a	Involved in the study	
☒	☐	ChIP-seq
☒	☐	Flow cytometry
☒	☐	MRI-based neuroimaging

Animals and other organisms

Policy information about studies involving animals; ARRIVE guidelines recommended for reporting animal research

Laboratory animals	Myzus persicae is a lower invertebrate and is not classified as a 'Laboratory animal'.

Wild animals	Study did not involve wild animals
Field-collected samples	Study did not involve samples collected directly from the field
Ethics oversight	Myzus persicae is a lower invertebrate and thus study of this species have no ethical requirements.

Note that full information on the approval of the study protocol must also be provided in the manuscript.
